# Piezoelectric‐Triboelectric Hybrid Nanogenerator for Energy Harvesting and Self‐Powered Sensing Applications

**DOI:** 10.1002/smll.202504626

**Published:** 2025-07-24

**Authors:** Syed Zubair Hussain, Vishwa Pratap Singh, Md Sazid Bin Sadeque, Shabnam Yavari, Gulnur Kalimuldina, Mustafa Ordu

**Affiliations:** ^1^ UNAM National Nanotechnology Research Center and Institute of Materials Science and Nanotechnology Bilkent University Ankara 06800 Türkiye; ^2^ Fiber Science Program Department of Human Centered Design College of Human Ecology Cornell University Ithaca NY 14853 USA; ^3^ Department of Mechanical and Aerospace Engineering School of Engineering and Digital Sciences Nazarbayev University Astana 010000 Kazakhstan

**Keywords:** energy harvester, hybrid nanogenerators, piezoelectricity, self‐powered sensing, triboelectricity

## Abstract

In the contemporary era, self‐powered sensors have gained significant attention, particularly in the domains of wearable devices, flexible electronics, healthcare monitoring devices, and the Internet of Things (IoT). Among the most promising technologies for mechanical energy harvesting are piezoelectric nanogenerators (PENGs) and triboelectric nanogenerators (TENGs), both of which convert ambient mechanical energy into electrical energy. However, the electrical output from either PENGs or TENGs alone is often insufficient to meet the power requirements of electronic devices. To address this limitation, the integration of piezoelectric and triboelectric effects into a single system has led to the emergence of piezoelectric‐triboelectric hybrid nanogenerators (PT‐HNGs). These hybrid systems represent a new class of energy harvesting devices capable of significantly enhancing energy conversion efficiency and output performance. This review provides a comprehensive overview of recent progress in developing PT‐HNGs, focusing on their underlying mechanisms, structural designs, coupling effects, performance optimization strategies, and diverse application potentials. It highlights the hybrid system's unique synergy and real‐world applicability, aiming to fill a critical gap in the literature. In addition, the review discusses the existing challenges, future directions, and prospects for the commercialization of PT‐HNG technology.

## Graphical abstract

This review highlights recent advancements in piezoelectric‐triboelectric hybrid nanogenerators (PT‐HNGs) for energy harvesting and self‐powered sensing. Emphasis is placed on material selection, device structures, coupling mechanisms, and performance optimization. The synergistic integration of piezoelectric and triboelectric effects enables improved output for real‐time applications in wearable technologies. Challenges, opportunities, and prospects of hybrid structures are also discussed to guide future research and practical implementation.

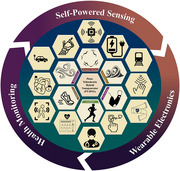

## 1 Introduction

In recent years, the burgeoning field of flexible electronics, portable, and wearable devices has attracted significant attention for research and practical applications.^[^ ^1^, ^2^ ^]^ These technologies hold immense promise but are often hindered by the limitations of traditional power sources, like batteries, which require frequent charging or replacement and contribute to the bulkiness of electronic systems.^[^ ^3^, ^4^, ^5^, ^6^ ^]^ This inconvenience restricts portability and integration into wearable items like clothing without compromising comfort. To deal with such challenges, researchers are trying to harvest energy from the environment, which appears to be an effective method for turning wasted mechanical energy into electricity.^[^ ^7^ ^]^ Human motion/activities,^[^ ^8^, ^9^, ^10^ ^]^ airflow,^[^ ^11^, ^12^, ^13^ ^]^ and water flow^[^ ^14^, ^15^, ^16^ ^]^ are all forms of mechanical energy sources that can be utilized to generate electrical energy.

Advancements in the field of nanotechnology have led to the development of innovative energy conversion devices, such as piezoelectric and triboelectric nanogenerators. Nanogenerators exhibit several benefits compared to conventional mechanical energy generators, especially their compact size, affordable cost, straightforward fabrication, and portability.^[^ ^17^, ^18^, ^19^, ^20^, ^21^, ^22^, ^23^, ^24^, ^25^ ^]^ Piezoelectric nanogenerators (PENGs) utilize the piezoelectric phenomena, wherein the application of mechanical force generates electric charges due to the polar crystalline structures of particular materials.^[^ ^8^ ^]^ Triboelectric nanogenerators (TENGs), on the other hand, operate on the coupled effects of contact electrification and electrostatic induction and can generate electrical potentials from mechanical energy.^[^ ^26^, ^27^, ^28^, ^29^, ^30^ ^]^ However, both TENGs and PENGs suffer from specific limitations. TENGs often exhibit poor performance under high humidity and may suffer from material degradation due to repetitive contact, while PENGs generally provide lower electrical output and are limited by material brittleness and mechanical fatigue.^[^ ^31^, ^32^ ^]^ These drawbacks reduce their reliability and efficiency in long‐term or real‐time applications. Hybrid PT‐HNGs have been introduced to overcome these issues by combining the strengths of both systems, leveraging the high surface charge density of TENGs and the internal polarization of PENGs to achieve enhanced, more stable, and consistent energy output.

**Figure 1:**
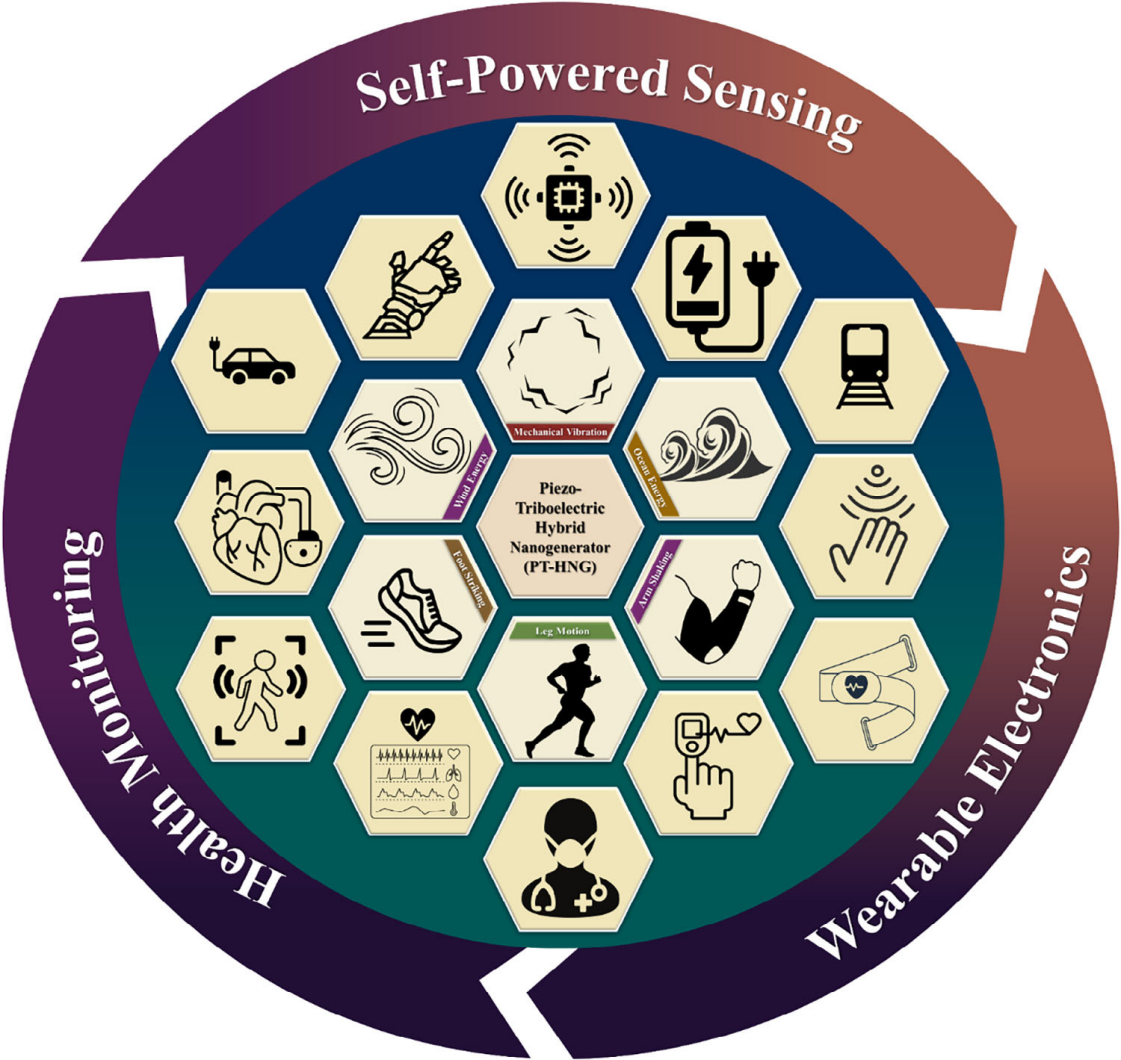
Potential applications of piezoelectric‐triboelectric hybrid nanogenerators (PT‐HNGs), with the central depiction of mechanical energy sources utilized for energy harvesting, surrounded by various applications, particularly in healthcare monitoring, self‐powered sensing, and wearable electronics.

Recently, hybridizing these two mechanisms, forming piezoelectric‐triboelectric hybrid nanogenerators (PT‐HNGs) has emerged as a novel and innovative approach to achieve real‐time energy harvesting and sensing applications. The idea of hybridizing PENGs and TENGs, as piezoelectric‐triboelectric hybrid nanogenerators (PT‐HNGs) has gained increasing demand owing to the potential for elevated output performances of nanogenerators.^[^ ^33^, ^34^, ^35^ ^]^ The hybrid structures aim to enhance the total power output by simultaneously harvesting mechanical energy and, at the same time, finding novel nanostructures and nanoarchitectures that further exploit triboelectric or piezoelectric conversions. The enhanced energy harvesting capabilities of PT‐HNGs result from the synergistic combination of PENGs and TENGs. TENGs harness electricity through surface charge interactions, while PENGs use piezoelectric effects originating from the crystalline structure.^[^ ^18^, ^36^ ^]^ Therefore, hybrid structures are capable of harvesting energy from both mechanical deformation and contact friction at the same time. This dual‐mechanism approach not only boosts performance but also offers unique benefits for real‐time and continuous operation in wearable and self‐powered electronic systems, which is currently a pressing research need. The improved power output of PT‐HNGs has been effectively applied in areas such as health monitoring, wearable electronics, and self‐powered sensing, as illustrated in **Figure** **1**. Recent progress has been made in developing flexible PT‐HNGs that can harvest biomechanical energy from various human movements while still being comfortable to wear. By allowing energy scavenging across an array of low‐frequency human motions, hybrid structures are one of the valid solutions for achieving flexibility while maintaining performance. Despite promising progress, the field lacks a comprehensive and application‐driven review that not only summarizes existing innovations but also contextualizes PT‐HNGs for real‐time usage scenarios. Numerous experimental and review works have been conducted to highlight the importance of PENGs and TENGs, along with fewer works on HNGs. Therefore, forming a comprehensive review in the field of PT‐HNGs will be useful, as the interest in hybrid structures is constantly increasing owing to the enhanced performance of such systems.

This review article provides a comprehensive analysis of the development of PT‐HNG technology, focusing on its basic operating principles and theoretical foundations. Furthermore, the study discusses significant methods to enhance electrical outputs, specific applications in self‐powered sensors and energy harvesters, current challenges, and potential solutions for introducing PT‐HNGs closer to being widely applied to next‐generation smart devices. The primary goal is to provide a foundation for seamlessly incorporating high‐performance, flexible, and sustainable energy harvesting systems into daily life. The novelty of this review lies in its focus on bridging PT‐HNG development with real‐time, wearable applications, aiming to guide future innovations and practical deployments.

## 2 Theoretical Foundations of the PENG and TENG for Charge Generation

The PENG devices work on the piezoelectric principle, which Pierre and Jacques Curie discovered in 1880.^[^ ^37^ ^]^ The material with no centrosymmetry generates the piezoelectric potential by applying mechanical force or deformation under an electric field (converse effect). Essential requirements are that the material should have one of the non‐centrosymmetric crystal structures, enabling dipole formation in 20 of 21 non‐centrosymmetric point groups, and often ferroelectric properties for enhanced response through poling, as seen in materials like lead zirconate titanate (PZT), barium titanate (BaTiO_3_), and potassium sodium niobate (KNN). Later on, in 2007, the first zinc oxide (ZnO) nanowires‐based nanogenerators were developed, showing the conversion of low‐amplitude mechanical stimuli to electrical signals.^[^ ^38^ ^]^ The governing equations for the piezoelectric effect are the constitutive relations:

(1) $$D_{i}=e_{ip}\;\varepsilon_{p}+\kappa_{ik}E_{k}$$

(2) $$\sigma_{p}=c_{pq}\;\varepsilon_{q}-e_{kp}E_{k}$$

where (*D_i_*) is the electric displacement, (*E_k_*) is the electric field, (σ_*p*_) and (ε_*p*_) are stress and strain tensors, (*e_ip_*) is the piezoelectric coefficient tensor, (*c_pq_*) is the elastic modulus tensor, and (κ_*ik*_) is the dielectric tensor. These coupled equations describe the interplay between mechanical deformation and electric field generation. The electrical output of PENGs results from the piezoelectric potential created by mechanical strain, which drives electron flow in an external circuit. The remnant displacement, $(\;D_{ri}=e_{ip}\;\varepsilon_{p}),$ quantifies the body and surface charges generated, leading to a potential drop across electrodes. PENGs can be modeled as a voltage source in series with a capacitor (CP) or a current source in parallel with CP, where the displacement current density, ( *J_D_* =  ∂*D*/∂*t*), depends on the strain rate and piezoelectric coefficient (d_ik_). The piezoelectric coefficients d_33_ (polarization along the poling axis due to stress in the same direction) and d_31_ (polarization due to perpendicular stress) quantify performance, with high d_33_ (≈600 pC/N in PZT, ≈400–500 pC/N in KNN) critical for efficient energy harvesting. Stability under mechanical and electrical loads, and phase boundaries like the morphotropic phase boundary (MPB) in PZT, further boost piezoelectricity by facilitating polarization rotation. Figures of merit (FOM) for PENG include (d_33_^2^/ɛ_r_) for off‐resonance power output, where low permittivity (ɛ_r_) reduces dielectric losses, and (k^2^Q_m_/ ɛ_r_) for resonance, where k (electromechanical coupling factor, ≈0.7 in PZT, ≈0.5 in BaTiO_3_) and (Q_m_) (mechanical quality factor) govern efficiency. The voltage constant (g_33_ = d_33_/ ɛ_0_ɛ_r_) and product (d_33_.g_33_) are vital for voltage generation, while energy density scales with (d_33_^2^ σ^2^/2ɛ_r_). Curie temperature (T_c_), e.g., ≈420°C for KNN, ≈200–350°C for PZT, ensures thermal stability. Due to biocompatibility, lead‐free materials like KNN and BaTiO_3_ are promising for wearable PENGs, though their lower (T_c_) and processing challenges require optimization. For instance, nanoscale dimensions in materials like ZnO nanowires reduce capacitance, boosting voltage output, though current remains limited. These principles guide material selection, structural design, and circuit optimization to achieve high‐performance PENGs for self‐powered IoT devices and wearable electronics applications. PVDF is one of the most popular piezoelectric polymer materials utilized in developing piezoelectric nanogenerators, which was first discovered in 1969.^[^ ^39^ ^]^ PVDF is a semi‐crystalline polymer with notable piezoelectric properties, characterized by coefficients around d_31_ = 20 pC/N and d_33_ ranging from 20 to 33 pC/N.^[^ ^39^, ^40^ ^]^ These properties are primarily influenced by crystalline phases, which include five different known polymorphs: α, β, γ, δ, and ε. The piezoelectric nature of PVDF is primarily due to its polar crystalline phases, the β and γ phases, especially with the β phase having the highest electric dipole moment among them. The piezoelectric nature or polarization depends on the conformational structure of PVDF, like the non‐centrosymmetric structure of inorganic materials like PbTiO_3_ and barium titanate (BTO). Thus, enhancing the β phase content in PVDF is a practical approach to boost its piezoelectric performance, making PVDF‐based materials highly suitable for piezoelectric nanogenerators.^[^ ^41^, ^42^ ^]^

**Figure 2:**
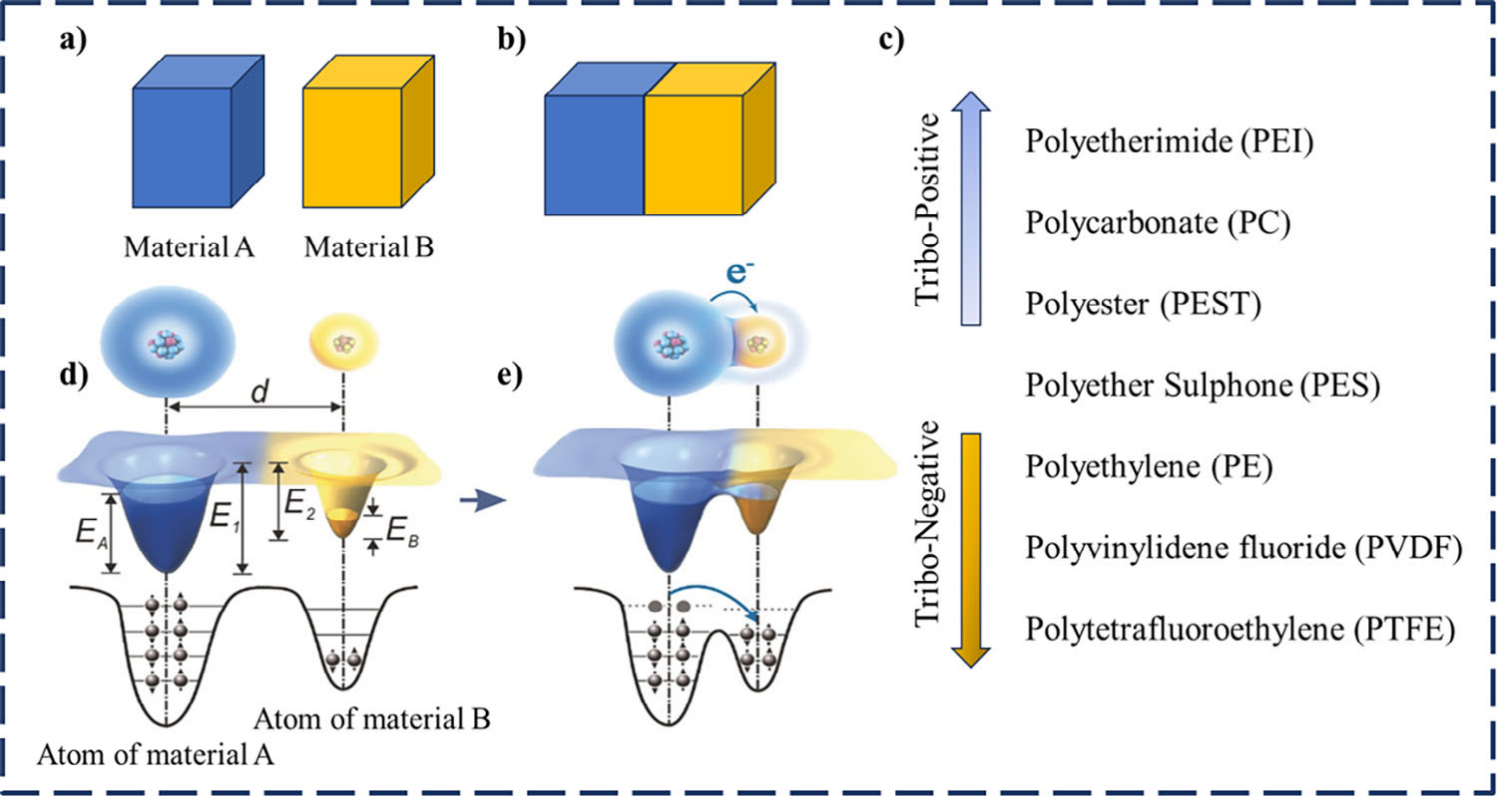
Mechanism of charge transfer in TENGs. a) Separated materials b) contact of materials and c) triboelectric series. Reprinted (Adapted) with permission.^[^ ^46^ ^]^ Copyright 2024, American Chemical Society. d) Work function of Kapton and PET, and e) Charge density of Kapton and PET. Reprinted (Adapted) with permission.^[^ ^45^ ^]^ Copyright 2022, American Chemical Society.

J Lowell and A.C. Rose‐Innes described the contact electrification process of transferring the charge when different materials contact each other and then separate.^[^ ^43^ ^]^ The different alignment of Fermi levels causes this resultant charge transfer. The charge transfer process in contact electrification can be understood with the help of the surface state model for metals and semiconductors. The Fermi‐Dirac distribution function, denoted as 𝑓, describes the probability of occupancy of electronic states at a given energy level *E* in a system of fermions at thermal equilibrium. Mathematically, it is expressed as

(3) $$f=\frac{1}{\mathrm{ex}p^{\left(E-E_{F}\right)/kT}+1}$$

where *E* is the energy of the state, *E_F_* represents the Fermi energy, 𝑘 is the Boltzmann constant, and *T* is the absolute temperature. This function indicates that the probability of an electron occupying an energy state decreases exponentially as the energy increases above the Fermi level. However, this model can be followed by metal‐metal and metal‐semiconductor as it originated from the band structure theory of semiconducting materials, which is not applicable to polymer and non‐crystalline materials. Contact electrification of polymer to polymer or polymer to non‐crystalline can be understood with the help of electron clouds that are localized explicitly in the molecular orbital or atom.^[^ ^44^ ^]^ **Figure** **2** shows that the atom can be considered or represented by the potential well having loosely bound electrons in the outer shell. The separation of both materials A and B is defined by a distance *d* between their electron clouds, with *E_A_* and *E_B_* as their electron energy levels and *E_1_* (<*E_A_*) and *E_2_* (<*E_B_*) as the escape potential energies. Before contact, electron transfer is halted by the local trapping effect of the potential wells. Upon contact between both materials, the electron clouds overlap, leading to a “screening” effect and transforming the initial single potential well into an asymmetric double‐well potential, making electrons hop from material A to B, as depicted in Figure 2. After materials A and B separate, the electrons transferred to material B remain due to the energy barrier *E_2_*, provided that the temperature does not change significantly or become excessively high. Thus, charge exchange results in positively charged material A and negatively charged material B. The electronegative (electron‐deficient) with a lower occupied level can easily accept the electron from the electropositive materials with the highest occupied level. Figure 2c shows the triboelectric series of selected triboelectric materials. Figures 2c,d illustrate polyimide (PA, commercially known as Kapton) and PET's energy band diagrams and triboelectric charge density. Due to its lower work function, Kapton donates electrons upon contact with PET, which has a higher work function, leading to a net positive charge on Kapton and a negative charge on PET after separation. Experimentally, Figure 2d shows that Kapton exhibits a slightly higher average triboelectric charge density than PET, with error bars indicating measurement variations.^[^ ^45^ ^]^

The electrostatic phenomena of materials can be understood with the help of the famous Maxwell's equations, which describe the core principles of electromagnetism. Gauss's Law for Electricity states that the electric field divergence relates to charge density. Gauss's Law for Magnetism shows that magnetic fields have no divergence, meaning magnetic monopoles don't exist. Faraday's Law indicates that a changing magnetic field induces an electric field. Ampère's Law, with Maxwell's addition, connects magnetic fields with electric currents and time‐varying electric fields. The electromagnetic system has two types of currents: conduction current and displacement current. Conduction current is due to the flow of electrons in conductors, whereas the displacement current is due to varying electric fields without the role of charge flow. So, the most crucial part is the inclusion of the displacement current in Ampere's aw to satisfy the conservation law of charge. It is well known that the PENG and TENGs are not subjected to any external electric field to generate energy, though they need mechanical stimuli. The polarization *P_s_* term is added in displacement *D* to explain the output of the PENG and TENG; the polarization *P_s_* is developed in the nanogenerator system due to mechanical stimuli in place of electric field (*E_Q_*) based dielectric polarization *P*.^[^ ^47^ ^]^ So, the modified displacement current density is given by Equation (2) as follows:

The total displacement current density is:

(4) $$J_{D}=\varepsilon \frac{\partial E}{\partial t}+\frac{\partial Ps}{\partial t}$$

In this equation, *J_D_* represents the total displacement current density, E is the electric field, ϵ is the permittivity of the dielectric material, and *Ps* is the polarization with time *t*.

**Figure 3:**
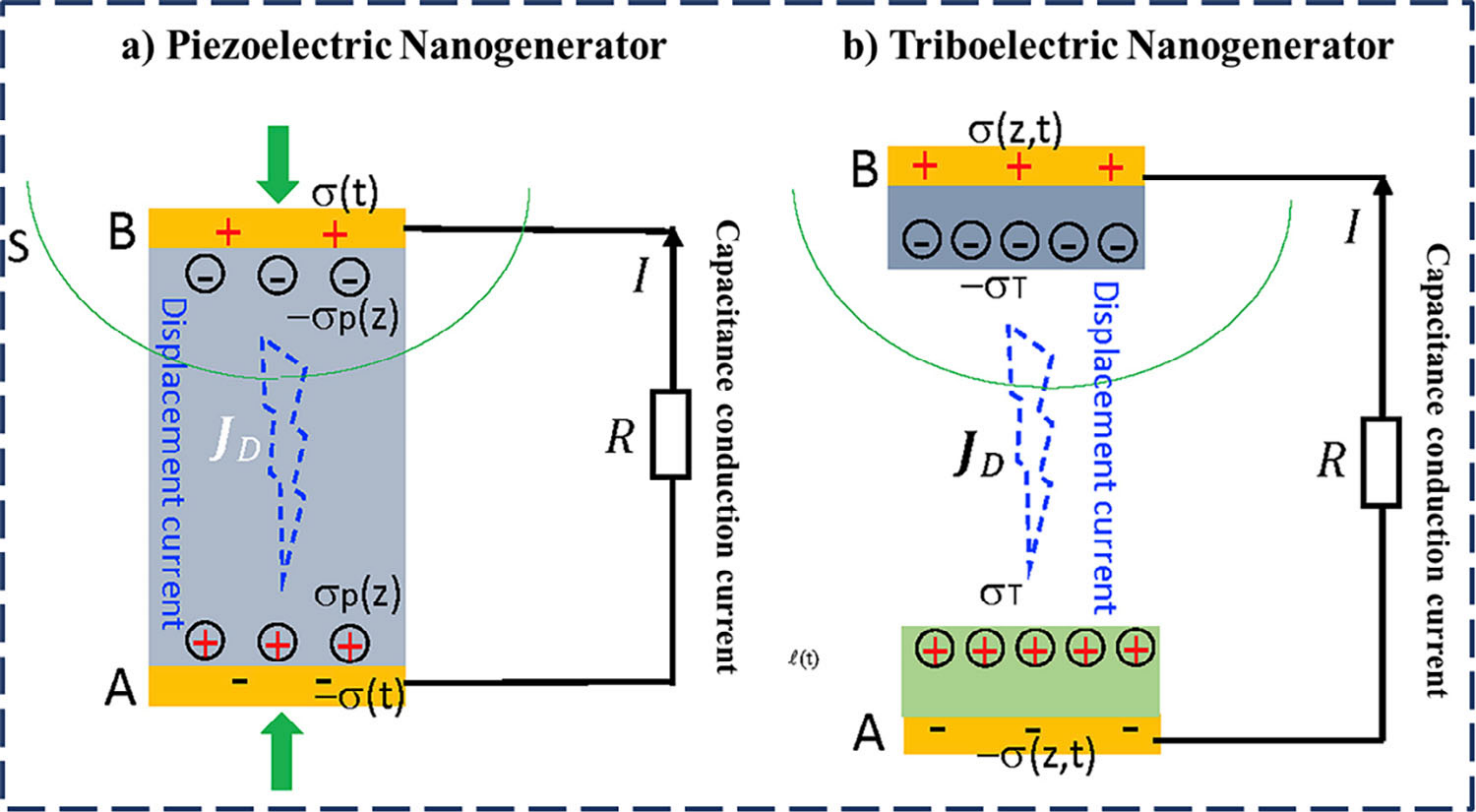
Working principles of a) a PENG and b) a TENG. Reprinted (Adapted) with permission.^[^ ^47^ ^]^ Copyright 2020, Elsevier.

The displacement current is an integral surface of J_D_.

(5) $$I_{D}=\int J_{D}\cdot ds=\frac{\partial Q}{\partial t}$$

where, *I_D_* is the displacement current, *Q* is the charge, and *ds is the surface area*. **Figure** **3**a shows that when the mechanical force is applied to the piezoelectric material having two electrodes on opposite layers, it tends to deform and eventually generates piezoelectric polarization, inducing a charge density *σ (t)* on the electrode. The expected outcome of PENG is given below by Equation 4:

(6) $$RA\;\frac{d\sigma }{dt}=\frac{z\left[\sigma_{P}-\sigma \right]}{\varepsilon }\;$$

where *R* is the external load, *A* is the area of the electrode, σ_*P*_ is the piezoelectric polarization charge density, σ is the surface charge density on the electrode, and z is the displacement or gap distance. Figure 3b shows the contact‐separation mode of a TENG gap distance *z* between these layers, along with the triboelectric surface charge density *σ_T​_(t)* and the electrode surface charge density *σ(z,t)*. The output current density *J_D_* ​ can be written as below by Equation (5):

(7) $$J_{D}=\sigma_{T}\;\frac{dH}{dt}\frac{\left(d_{1}\varepsilon_{0}/\varepsilon_{1}+d_{2}\varepsilon_{0}/\varepsilon_{2}\right)}{{\left(d_{1}\varepsilon_{0}/\varepsilon_{1}+d_{2}\varepsilon_{0}/\varepsilon_{2}+z\right)}^{2}}$$

The final output equation can be written as:

(8) $$RA\;\frac{d\sigma \left(z,t\right)}{dt}=\frac{z\sigma_{T}}{\varepsilon_{0}}\;-\;\sigma \left(z,t\right)\left[\frac{d_{1}}{\varepsilon_{1}}+\frac{d_{2}}{\varepsilon_{2}}+\frac{z}{\varepsilon_{0}}\right]$$

where *Q* is charge, *H(t)* is the time‐dependent separation distance between the two dielectric layers in the contact‐separation mode, *ϵ_0,_ ϵ_1_​* and *ϵ_2_* are the permittivity of the free space and respective dielectric materials, *d_1_* and *d_2_* are the thicknesses of the dielectric layers, *R* is the resistance of the external load, *A* is the area of the electrode and *z* is the gap distance between the dielectric layers.

### 2.1 Coupling of Piezoelectric and Triboelectric Effects for Enhanced Energy Harvesting Performance

The performance of the nanogenerators depends on the materials, device shape/geometry, and modes.^[^ ^48^, ^49^ ^]^ Coupling the TENG and PENG enhances the performance of the overall device multiple times. The TENG performance depends on the surface charge density of the material.^[^ ^50^ ^]^ Coupling the PENG along with TENG can provide more surface charge density that can enhance performance, as the piezoelectric effect induces an internal electric field that promotes charge separation at the interface, thereby amplifying triboelectric output. Simultaneously, the mechanical deformation during triboelectric contact improves dipole alignment in the piezoelectric material, boosting its polarization.^[^ ^51^ ^]^ Furthermore, the strain in the piezoelectric material will produce a charge on the surface due to mechanical stimuli.^[^ ^52^ ^]^ The combination of piezoelectric and triboelectric effects does not simply yield an additive improvement in output, but rather, it is a synergistic enhancement that arises from the interaction of the charge of the materials at the surface.^[^ ^53^ ^]^ Several research studies have shown that the hybridizing piezoelectric and triboelectric effects accumulate tribo‐charge on the triboelectric component and establish an electric field that influences the polarization of the piezoelectric material.^[^ ^54^ ^]^ When the polarization direction in the piezoelectric layer aligns with this electric field, the hybrid device's output is increased; if misaligned, the output would be reduced. In typical designs, the triboelectric layer is crafted from friction materials with distinct surface potentials to promote charge transfer. Interestingly, research has demonstrated that in hybrid nanogenerators, piezoelectric materials like PVDF play a crucial role in enhancing triboelectric charge transfer. When integrated into the triboelectric layer, the intrinsic dipole moment of PVDF modifies the surface potential, facilitating charge transfer even between identical materials. Additionally, the piezoelectric effect can induce an asymmetric charge distribution, which enhances the triboelectric output by preconditioning the contact surfaces with complementary charges before the contact‐separation cycle. This synergy between piezoelectric and triboelectric mechanisms significantly improves the overall energy‐harvesting efficiency of hybrid nanogenerators.^[^ ^55^ ^]^ This phenomenon was experimentally validated in a two‐electrode hybrid nanogenerator design using aligned PVDF fiber mats.^[^ ^56^ ^]^ Both mats were poled in the same axial direction (e.g., top‐to‐bottom polarization). Under compression, the piezoelectric effect generated opposing charges on the contacting surfaces: the upper mat's bottom surface developed a negative charge, while the lower mat's top surface became positive. This intrinsic piezoelectric polarization created a potential difference, driving charge transfer between the electrodes. Critically, this piezoelectric‐induced asymmetry amplified the triboelectric output by preconditioning the surfaces with complementary charges prior to contact‐separation cycles.^[^ ^57^ ^]^ Utilizing the hybrid concept, enormous research has been published on the PVDF/ZnSnO_3_ nanocube/ZnO nanosheet layer functions as both a piezoelectric and triboelectric element in combination with the PA6 film.^[^ ^58^ ^]^ The negative piezoelectric charges generated by PVDF and ZnO nanosheets lower the surface potential on the PVDF/ZnSnO_3_/ZnO film. This reduction boosts the contact surface potential difference between the PA6 film and the PVDF/ZnSnO_3_/ZnO layer, leading to a notable increase in the transfer of triboelectric charges during contact. As a result of this piezoelectric‐charge‐inducing effect, the triboelectric output power density of the nanogenerator is enhanced from 110 to 1800 mW m^−^^2^.^[^ ^58^ ^]^

**Figure 4:**
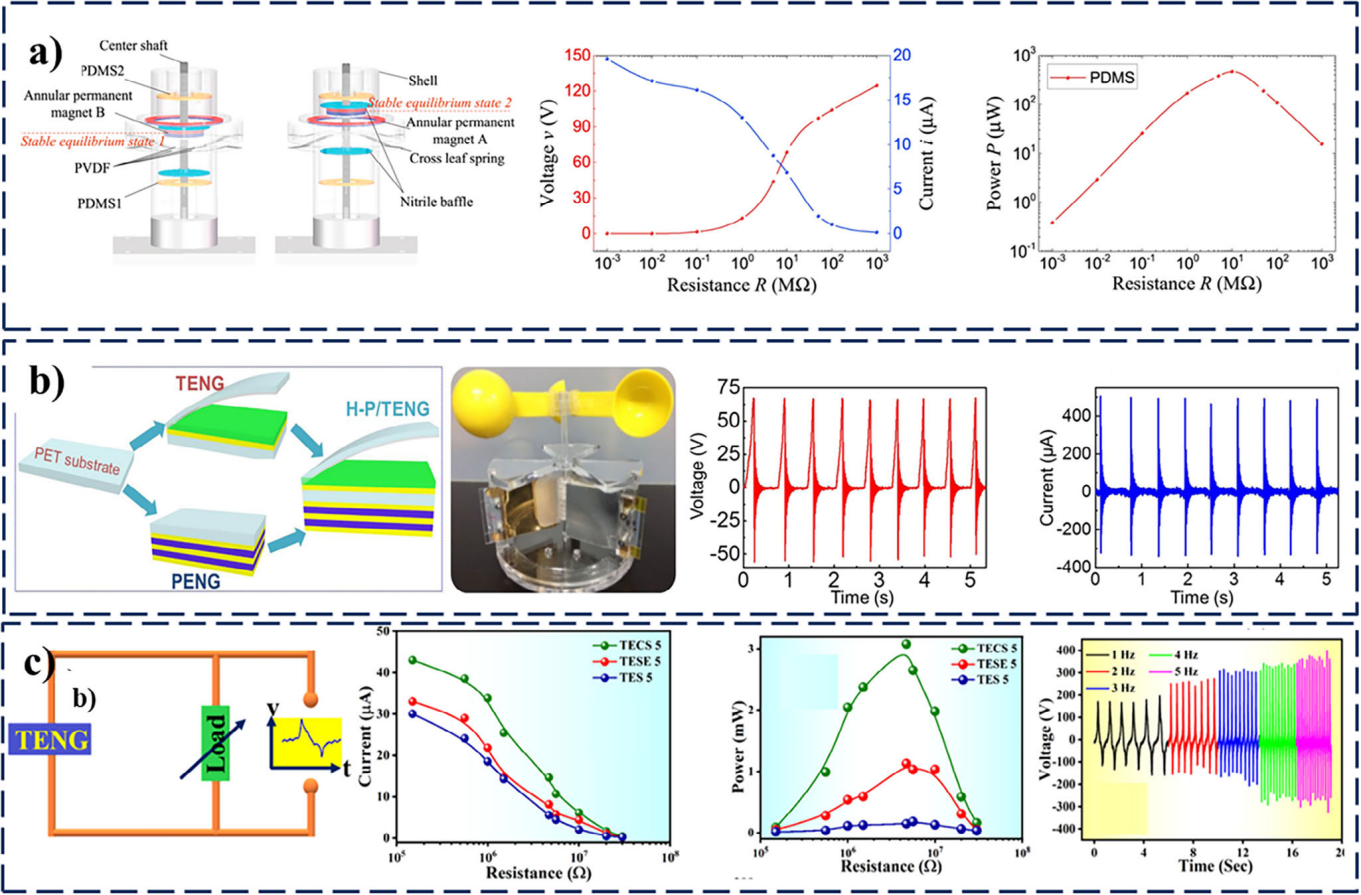
Coupling of Piezoelectric and Triboelectric Effects. a) PVDF/PDMS‐based PT‐HNG as a stable linear generator. Reprinted (adapted) with permission.^[^ ^59^ ^]^ Copyright 2019, Elsevier. b) poled PVDF‐TrFE, PTFE, and Al layers as PT‐HNG for high performance. Reprinted (adapted) with permission.^[^ ^60^ ^]^ Copyright 2019, Elsevier. c) CsPbI_3_ reinforced in PVDF as PT‐HNG for humidity sensor. Reprinted (adapted) with permission.^[^ ^62^ ^]^ Copyright 2024, American Chemical Society.

The study was done as shown in **Figure** **4**a, demonstrates the excellent performance of a PT‐HNG using PVDF, polydimethylsiloxane (PDMS), and nitrile rubber integrated into a baffle structure, achieving a *V_OC_* of ≈138 V and a power density of ≈878 mW m^‐^^3^.^[^ ^59^ ^]^ The innovative design effectively harnesses mechanical energy by combining the piezoelectric and triboelectric properties of the materials, significantly enhancing energy harvesting efficiency and offering the potential for low‐power electronic applications. Building on the concept of PT‐HNG, another study utilizing poled polyvinylidene fluoride‐co‐trifluoroethylene (PVDF‐TrFE), polytetrafluoroethylene (PTFE), and Al layers has demonstrated an impressive performance, achieving *V_OC_* of ≈210 V and *I_SC_* of ≈395 mA, corresponding to a power density of ≈6 mW cm^‐^^2^ shown in Figure 4b.^[^ ^60^ ^]^ This indicates that hybrid systems can significantly enhance energy harvesting capabilities by carefully selecting and combining piezoelectric and triboelectric materials. PVDF‐TrFE was employed to fabricate PENG, highlighting remarkable performance; however, its output falls short compared to the exceptional efficiency achieved by PT‐HNG systems. While the pure piezoelectric approach leverages the intrinsic properties of PVDF‐TrFE, PT‐HNG systems combine both piezoelectric and triboelectric effects and show significantly enhanced energy output, resulting in surpassing the performance of pure PENG.^[^ ^61^ ^]^ The enhancement of the surface charge density was demonstrated for achieving excellent harvesting performance of PT‐HNG by reinforcing CsPbI_3_ in PVDF polymer for application as a highly sensitive humidity sensor.^[^ ^62^ ^]^ The performance of the device was shown in three modes. Out of all modes, the contact‐separation mode exhibits *V_OC_* = 377 V and an *I_SC_* = 43 µA for a 10 mm separation distance between two dielectric layers, as shown in Figure 4c. The device exhibits strong protection against humidity due to the hydrophobic nature of the PVDF matrix and the uniform dispersion of CsPbI_3_ nanocrystals, which prevent moisture penetration. Also, it maintains stable output even at 90% relative humidity, ensuring its robust humidity resistance and operational stability in moist environments. In contrast, with the same material, CsPbI_2_Br reinforced in PVDF shows the triboelectric *V_OC_* = 225 V, *I_SC_* = 24.3 µA, and the power density of 1.54 W m^−2^ at 40 MΩ.^[^ ^63^ ^]^ When comparing the performance of PENG and TENG systems using nearly identical materials, PT‐HNG consistently outperforms both, demonstrating its superior efficiency. **Table** **1** shows the performance of the PT‐HNG of the current research in the last few years. In three‐electrode devices, PENGs and TENGs are seamlessly integrated to enhance energy harvesting through two primary electrodes that collect charges and a third electrode serving as a shared reference or ground to optimize charge flow. For instance, in a system using silicon rubber with PTFE particles or silver‐coated spheres paired with PZT powder, Electrode 1 (top) interfaces with the triboelectric silicon rubber layer to generate charges via contact‐separation, while Electrode 2 (bottom) captures piezoelectric charges from the highly piezoelectric PZT powder.^[^ ^64^ ^]^ The third electrode, shared between PENG and TENG, minimizes charge leakage and balances potential, resulting in an impressive output of 600 V, 17 µA, and a power density of 1.11 W m^−2^. Similarly, a configuration combining PDMS with carbon nanotubes (CNTs) and graphite nanoparticles alongside PVDF with CNTs and BTO nanofibers employs Electrode 1 for triboelectric charge induction, Electrode 2 for piezoelectric charge collection, and Electrode 3 to enhance charge transfer, achieving 160 V and a power density of 2.22 W m^−2^.

**Table 1:** Performances of PT‐HNGs,. V_max_ is for the open circuit, and I_max_ for short circuit conditions. PVA: polyvinyl alcohol.

Device form/ Working area	Materials	V_max_	I_max_	Frequency	Peak Power/ Power density	Refs.
Woven strips 6 × 6 cm^2^	Silicon rubber with PTFE & Ag‐coated microspheres	600 V	17 µA	1–3 Hz	1.11 W m^−2^	[64]
Capsule 223 × 223 × 205 mm^3^	Polylactic Acid, Polymaker‐Tough/Al‐Cu	20.8 V	−	4.5 Hz	70 µW	[65]
Composite film 13.3 × 13.3 × 0.3 mm^3^	PVDF nanofibers/PDMS Nylon fabric film	88 V	5.85 µA	1 Hz	286 mW m^−2^	[66]
3D Printed substrate 25 × 25 × 0.4 mm^3^	PDMS‐BaTiO_3_ coated steel substrate	405 V	38 µA	5 Hz	2.2 mW cm^−2^	[67]
Composite film 2.5 × 2.5 cm^2^	PDMS/Ba(Zr_0.2_ Ti_0.8_)O_3_–0.5(Ba_0.7_Ca_0.3_) TiO_3_–BCT and PVA films	127.1 V	66.6 mA m^−2^	1 Hz	7.5 W m^−2^	[68]
Composite film 3 × 3 cm^2^	γ‐GC/CS & PTFE, Al tape	78.81 V	64 µA	4 Hz	79 µW cm^−2^	[69]
Composite film 2.5 × 2.5 cm^2^	Cu_2_O‐doped/0.3Ba_0.7_Ca_0.3_TiO_3_‐0.7BaSn_0.1_Ti_0.9_O_3_ NPs/PDMS	176.41 V	0.45 µA	−	168.1 mW m^−2^	[70]
Composite film 3 × 3 cm^2^	BaTiO_3_ nanorods in chitosan matrix, PTFE	111.4 V	21.6 µA cm^‐^^2^	0.55 Hz	756 µW cm^−2^	[71]
Composite film 25 × 25 mm^2^	Nanoporous film of PVDF/BaTiO_3_ Electrospun Fibers	444 V	19.02 mA m^−2^	4 Hz	0.4 W m^−2^	[72]
Composite film 1.5 × 1.5 cm^2^	BT‐1500/PDMS composite film	100 V	2 µA	−	0.89 W m^−2^	[73]

Another effective three‐electrode system utilizes PDMS with graphite nanoparticles and PVDF‐TrFE combined with Ag nanowires to maximize energy harvesting. Here, Electrode 1 interfaces with the PDMS‐based triboelectric layer to induce charges through contact‐separation, while Electrode 2 collects piezoelectric charges from the PVDF‐TrFE and Ag nanowire composite, which benefits from enhanced conductivity. The third electrode ensures efficient charge management by reducing losses and stabilizing potential, leading to a performance of 190 V and a power output of 16.46 µW.^[^ ^74^ ^]^ In all cases, mechanical force drives piezoelectric charge generation between Electrodes 1 and 2, while triboelectric interactions generate additional charges, with the third electrode playing a pivotal role in boosting overall efficiency by optimizing charge flow and minimizing leakage in these hybrid PENG‐TENG systems.

## 3 Structure of Piezoelectric‐Triboelectric Hybrid Nanogenerators

The choice between two‐electrode and three‐electrode modes depends on specific application requirements, including desired output performance, device complexity, and fabrication considerations. Both configurations operate on nearly the same principle, with the primary difference being the number of electrodes used. Understanding each configuration's operational principles and advantages enables the design of PT‐HNGs tailored to meet diverse energy harvesting needs. Both schemes are discussed in the following section and numerically and experimentally covered in numerous works.

### 3.1 Two‐Electrode Scheme

**Figure 5:**
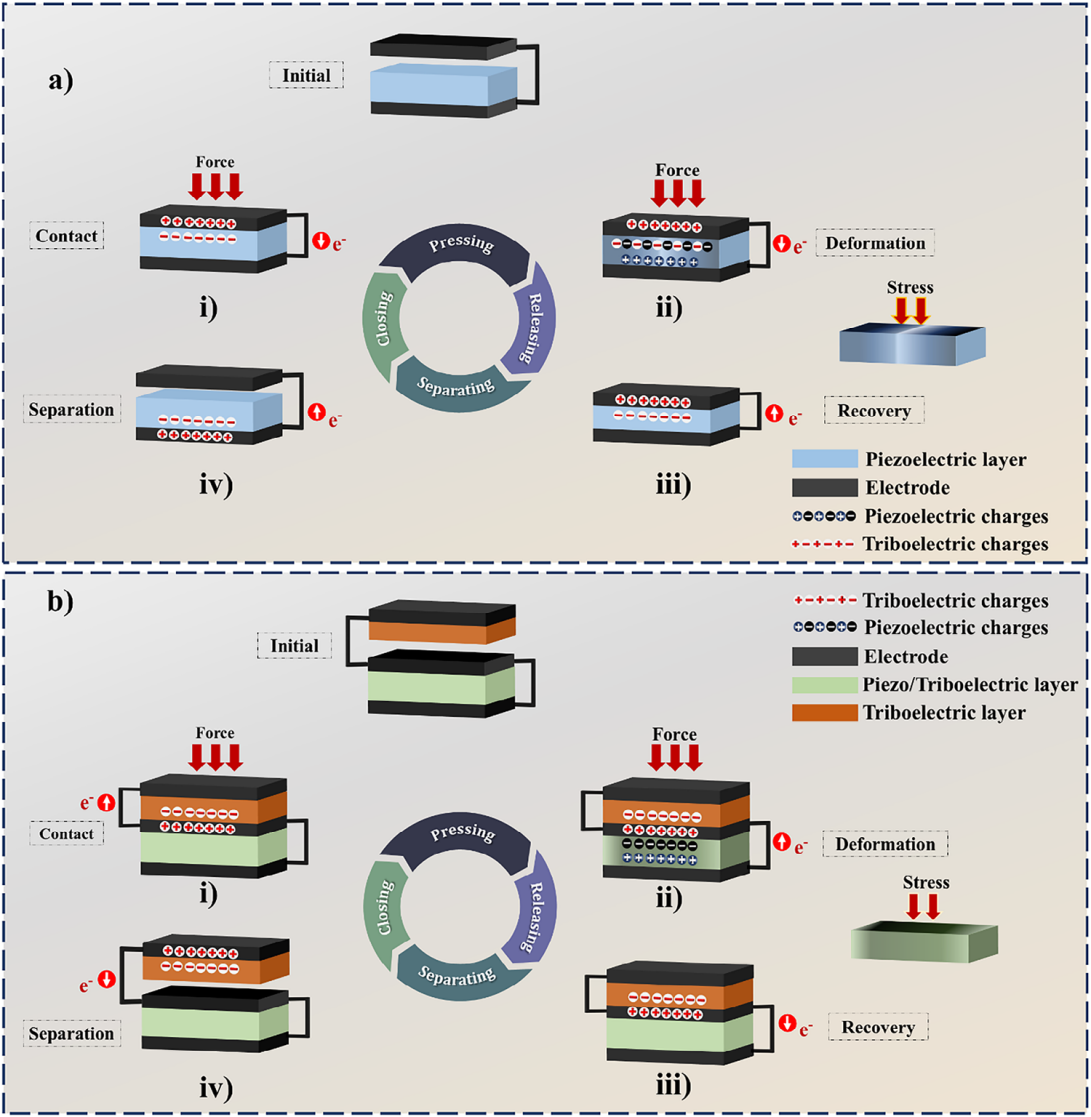
PT‐HNGs systems. Working mechanism of a) two‐electrode module and b) three‐electrode module.

The two‐electrode structure involves a single functional layer with both piezoelectric and triboelectric properties sandwiched between two electrodes. This structure requires a spacer between one electrode and the functional layer, allowing a contact‐separation cycle for a triboelectric effect. Initially, while force is applied externally, the electrode at the top approaches the functional layer until contact is made (**Figure** **5**a‐i). During this stage, due to triboelectrification, electrons are transferred from the top electrode to the bottom electrode through the external circuit. While increasing the pressure (Figure 5a‐ii), the piezoelectric layer undergoes a stimulated piezoelectric effect, electrons flow from the top electrode to the bottom electrode, reinforcing the electron flow initiated by the triboelectric effect via the external circuit. Upon release of the external force (Figure 5a‐iii), the piezoelectric polarization diminishes, and the electrons return from the bottom electrode to the top electrode to restore electrostatic equilibrium. Due to this, a piezoelectric current is induced in the opposite direction. During the final phase (Figure 5a‐iv), as the top electrode separates further from the piezoelectric layer, the electrostatic induction between them decreases. This leads to electrons flowing from the bottom electrode to the top electrode, opposite to the initial triboelectric phase, completing the full energy generation cycle.

**Figure 6:**
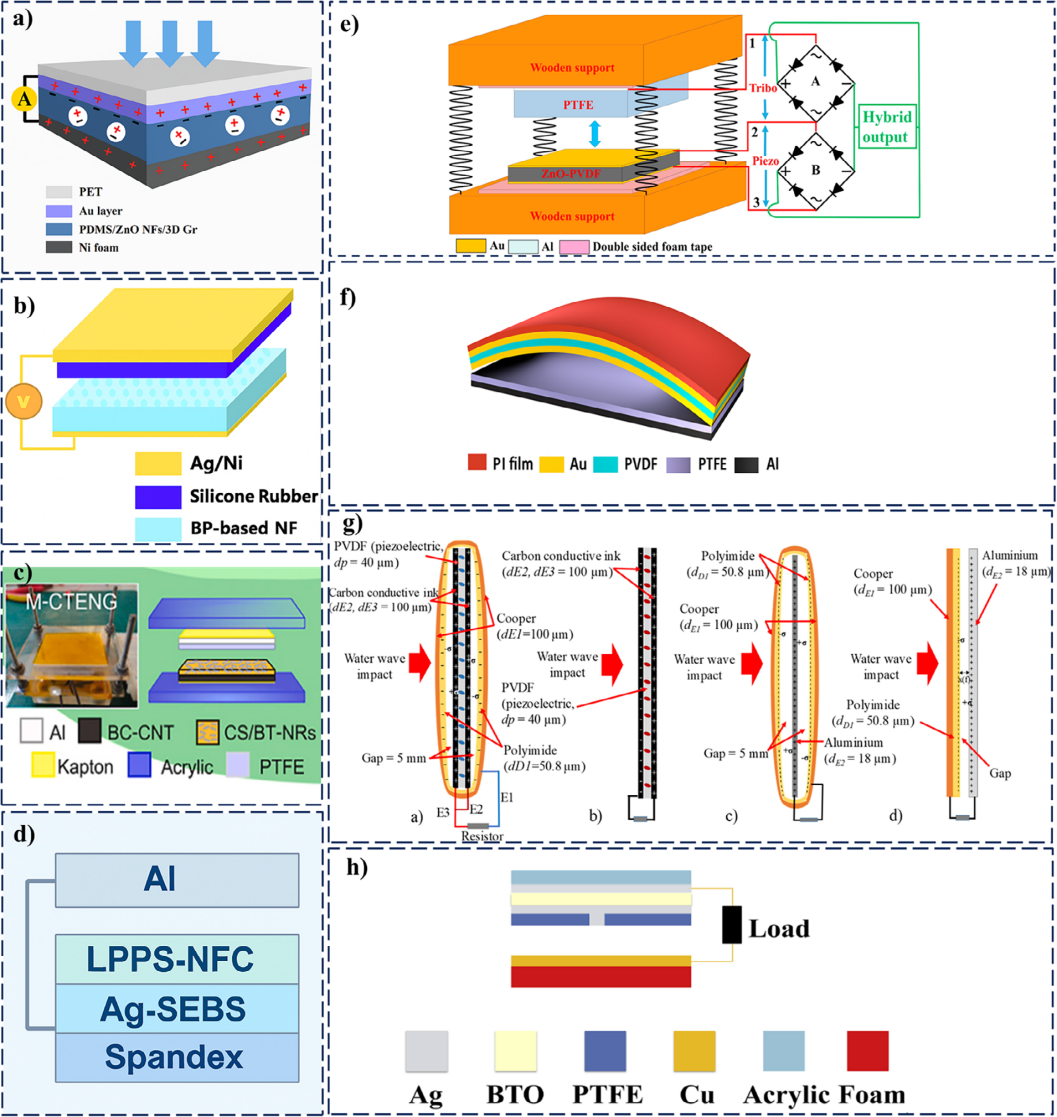
Schematic illustration of two and three‐electrode PT‐HNG based on a) PDMS/ZnO NFs/3D Gr/Ni foam. Reprinted (Adapted) with permission.^[^ ^75^ ^]^ Copyright 2018, American Chemical Society. b) BCZT/PVDF‐HFP composite nanofibers (BP‐based NFs). Reproduced (Adapted) with permission.^[^ ^76^ ^]^ Copyright 2019, The Royal Society of Chemistry. c) BaTiO_3_‐Nanorods/Chitosan, Reprinted (Adapted) with permission.^[^ ^71^ ^]^ Copyright 2021, Elsevier. d) Stretchable, Breathable, and Stable Lead‐Free Perovskite/Polymer‐based PT‐HNG. Reprinted (Adapted) with permission.^[^ ^77^ ^]^ Copyright 2022, John Wiley and Sons. e) Schematic structure and electro‐circuit diagram of a ZnO nanorod/PVDF‐PTFE based PT‐HNG. Reprinted (Adapted) with permission.^[^ ^78^ ^]^ Copyright 2018, Elsevier. f) Diagram of an arc‐shaped PVDF‐PTFE‐based PT‐HNG. Reproduced (Adapted) with permission.^[^ ^79^ ^]^ Copyright 2015, Springer Nature. g) 2D schematic using three for improving ocean wave impact energy harvesting. Reprinted (Adapted) with permission.^[^ ^15^ ^]^ Copyright 2020, Elsevier. h) Schematic diagram of a PT‐HNG with point contact between two electrodes. Reprinted (Adapted) with permission.^[^ ^22^ ^]^ Copyright 2020, Elsevier.

Following the hybrid two‐electrode configuration, a PDMS/ZnO nanoflakes/3D graphene (PDMS/ZnO NFs/3D Gr) heterostructure was reported in (**Figure** **6**a).^[^ ^75^ ^]^ The top electrode was made of gold (Au) deposited on a poly(ethylene terephthalate) (PET) substrate, establishing a positive piezoelectric potential when a vertical force is applied. The bottom electrode, including the functional layer, comprises the PDMS/ZnO NFs/3D Gr/Ni foam structure. When the vertical force compresses the device, the top electrode (Au/PET), the bottom electrode and functional layer (PDMS/ZnO NFs/3D Gr/Ni foam) come into contact, allowing charge transfer and establishing a potential difference. The electrodes are separated upon removing the external force, and the potential difference enables electrons to flow from the bottom electrode to the top electrode through an external circuit. Combining the piezoelectric effect from the ZnO NFs and the triboelectric effect from the PDMS layer enhances the overall output performance of the energy harvester. The 3D graphene structure provides a continuous pathway for charge transfer, improving the device's efficiency. The power density of this hybrid nanogenerator was measured to be 6.22 mW cm^−2^. Another two‐electrode PT‐HNG utilizing composite nanofibers and silicone rubber simultaneously exhibits piezoelectric and triboelectric properties.^[^ ^76^ ^]^ The device contained four distinct layers (Figure 6b), with one (silicone rubber) functioning as an additional friction component and positioned between the upper electrode and the piezo‐triboelectric functional layer (Ba_0.85_Ca_0.15_)(Zr_0.1_Ti_0.9_)O_3_ (shortly as BCZT) nanoparticles incorporated polyvinylidene fluoride (PVDF) nanofiber network used as the piezoelectric layer. Silicon rubber exhibits a negative charge during the triboelectric process due to its higher electronegativity than PVDF, while PVDF receives positive charges. Ultimately, the generator provided a final output power density of 162 mW m^−2^. A two‐electrode PT‐HNG device was designed with BaTiO_3_ nanorods (BT‐NRs) embedded in a chitosan matrix to maximize the output (Figure 6c).^[^ ^71^ ^]^ The structure includes BT‐NRs at an optimal 7 wt% for enhanced piezoelectric performance, combined with the triboelectric properties of chitosan. The electrodes were crafted from a bacterial cellulose/carbon nanotube composite, achieving high contact efficiency, and the device's structural frame features an adjustable gap supported by metal springs. Kapton tape ensured the protection and wire stabilization of the structure. Functionally, the nanogenerator converts mechanical energy into electrical energy, producing an open‐circuit voltage of 111.4 V and a power density of 756 µW cm^−2^. When integrated with a self‐charge pumping module, the output performance quadrupled, reaching 247.2 V and 1568 µW cm^−2^. This innovative design demonstrates a highly efficient energy‐harvesting solution for diverse applications.

Another flexible PT‐HNG following two electrode modules exhibiting 2.34 W m^−2^ electrical output used spandex as a substrate and printed silver‐SEBS ink onto it for stretchable conductors (Figure 6d).^[^ ^77^ ^]^ The conductive textile is then electrospun with a mixture of polyvinylidene‐co‐hexafluoropropylene (PVDF‐HFP) and tricesium nonabromodibismuthate(III) (Cs_3_Bi_2_Br_9_), incorporating perovskites into nanofibers. Styrene Ethylene Butylene Styrene block copolymer (SEBS) microspheres were introduced as elastic binder during electrospinning for enhanced stretchability and hydrophobicity. The hybrid device, designed for harvesting mechanical energy from biomechanical movements, incorporates top electrodes fabricated from aluminum (Al) or conductive fabric separated by a spacer. **Tables** **2** and **3** summarize several recent works of PT‐HNG based on the two and three‐electrode configurations of PT‐HNGs, respectively.

**Table 2:** Examples of the two‐electrode configuration of PT‐HNGs.

Piezoelectric constituents	Triboelectric constituents	Output voltage	Output current	Output power	Refs.
Chitosan & BaTiO_3_	Chitosan & PTFE	247.2 V	36.7 µA/cm^2^	1568 µW cm^−2^	[71]
PVDF	Ni/Cu‐coated PET & PVDF	210 V	45 µA	2.1 mW	[80]
BaTiO_3_	PDMS	280 V	5.6 µA	0.04 mW cm^−2^	[81]
ZnO	PDMS & MW‐ CNTs & ZnO	400 V	30 µA	–	[82]
Ca‐doped Ba(Zr_0.2_Ti_0.8_)O_3_	PDMS	500 V	34 µA	23.6 W m^−2^	[83]

**Table 3:** Three‐electrode configuration of PT‐HNGs.

Piezoelectric constituents	Triboelectric constituents	Output voltage	Output current	Output power	Refs.
PVDF	Cellulose, MoS_2_ & PI	50 V	30 nA	0.18 mW cm^−2^	[84]
PVDF, CNT & BTO	PDMS, CNTs & Graphite	160 V	–	2.22 W m^−2^	[85]
PVDF	Acrylic & Al	180 V	5.3 µA	127 µW	[86]
PVDF‐TrFE & Ag nanowire	PDMS & Graphite	190 V	–	16.46 µW	[87]
PZT	Silicon rubber with PTFE & Ag‐coated microspheres	600 V	17 µA	1.11 W m^−2^	[64]

### 3.2 Three‐Electrode Scheme

The three‐electrode structure module introduces functional piezoelectric and triboelectric layers separately. Depending on the material pairing, the piezoelectric layer can also act as a triboelectric layer. For example, PVDF, being both piezoelectric and triboelectric, can participate in dual‐mode energy conversion.^[^ ^88^ ^]^ Its role as a positive or negative triboelectric material depends on the counterpart material in contact. This configuration typically includes an intermediate electrode placed between the piezoelectric and triboelectric layers. The triboelectric layers operate in a contact‐separation mode, while the piezoelectric layer is activated under mechanical stress. When an external force is applied, the upper triboelectric layer (which may be pre‐rubbed to enhance surface charge density) comes into contact with the middle electrode (Figure 5b‐i). This contact induces a triboelectric effect, wherein electrons are transferred based on the triboelectric polarity of the layers. Negative charges accumulate on the triboelectric layer, while corresponding positive charges are induced on the middle electrode. This results in electrons flowing from the middle electrode to the top electrode through the external circuit. As the applied force increases (Figure 5b‐ii), the device compresses further and activates the piezoelectric layer beneath the middle electrode. The deformation generates piezoelectric charges, and electrons flow from the middle electrode to the bottom electrode via electrostatic induction. When the external force is released (Figure 5b‐iii), the piezoelectric layer begins to recover its original shape, leading to a redistribution of internal charges. Consequently, electrons flow back from the bottom electrode to the middle electrode, restoring the charge equilibrium. In the final stage (Figure 5b‐iv), the electrostatic induction decreases as the top triboelectric layer separates from the middle electrode. This leads to electron flow from the top electrode to the middle electrode, completing the triboelectric cycle with a direction opposite to the initial contact.

A PT‐HNG structure operating on a three‐electrode module was reported by integrating ZnO‐PVDF nanocomposite film with PTFE.^[^ ^78^ ^]^ (Figure 6e). Au and Al layers act as electrodes that collect the electrical outputs generated by the triboelectric and piezoelectric effects. A 75 nm Au film was deposited above and below the composite film, and an aluminum layer of thickness 100 nm was coated above the PTFE film. Copper (Cu) wires were attached to the metal electrodes using silver (Ag) paste to facilitate the electrical connections. Although the triboelectric interface is physically between the PTFE and the thin gold (Au) layer deposited on the ZnO‐PVDF composite, the triboelectric output is effectively governed by the PTFE‐ZnO‐PVDF combination. This is due to the enhanced surface roughness and polarizability of the ZnO‐PVDF film, which influences the charge generation at the Au interface. In contrast, the piezoelectric output is generated from the mechanical stress applied to the PVDF film. The outputs from the electrodes are processed through bridge rectifiers to measure the combined electrical output.

An innovative arc‐shaped structured high‐output PT‐HNG was designed, comprising a piezoelectric top layer made of PVDF sandwiched between Au electrodes, and a triboelectric bottom layer consisting of a PTFE film (active layer) backed by an Al electrode (Figure 6f).^[^ ^79^ ^]^ The Al electrode, positioned beneath the PTFE triboelectric active layer, served as a shared driving electrode for both piezoelectric and triboelectric outputs. When an external mechanical force was applied to the device, the PVDF layer experienced compressive deformation, generating a positive piezoelectric potential between the Au1 and Au2 electrodes. This induced electron flow from Au2 to Au1, producing a piezoelectric current. Simultaneously, the top Au electrode came into contact with the PTFE layer, initiating a triboelectric effect due to the electron affinity difference between PTFE and Au. This contact resulted in electron transfer to the PTFE surface, leading to electrostatic charge accumulation. Upon release of the applied force, the piezoelectric and triboelectric output voltages reached their maxima, and the combined interaction of both mechanisms contributed to the total current output. This hybrid structure successfully integrates the advantages of both energy harvesting methods, enabling enhanced mechanical‐to‐electrical energy conversion during each press‐and‐release cycle.

Following three electrode schemes, a hybrid structure was reported^[^ ^15^ ^]^ (Figure 6g), which illustrates that two carbon conductive ink electrodes (E2 and E3) were printed on both sides of a flexible PVDF piezoelectric layer, and an additional electrode (E1) was insulated on the opposite side using a Kapton tape. E1 functioned as the second electrode connected to an external load for electrical characterization, while E2 served as a grounded triboelectric material and the second electrode for the piezoelectric layer. The electrodes functioned by utilizing the triboelectric effect (from E2 and E3) and the piezoelectric effect (from the PVDF layer). The piezoelectric layer created an electrical charge due to deformation when the structure was subjected to mechanical energy from ocean waves. At the same time, the triboelectric materials generated an additional charge through contact and separation. The device was specifically designed and tested to harvest energy from breaking water waves, demonstrating stable performance under continuous wave impact at frequencies between 0.7 and 3 Hz. It maintained high output (up to 229.31 V and 140.93 µA) and durability during prolonged exposure by being insulated with polyethylene packaging. These results confirm its effectiveness and reliability in harsh, water‐based environments for marine energy harvesting applications.

The PT‐HNGs use a three‐electrode design to harvest energy from both triboelectric and piezoelectric effects without having a direct connection between them. Due to this, the performance of both piezo and triboelectric functional layers can be enhanced in this configuration. However, it requires a common electrode and two separate external circuits to collect energy from both sources individually. Although the separation improves efficiency, it also makes the device bulkier due to the extra components needed for the circuits and the shared electrode. As a consequence of the challenge, researchers put their efforts toward optimizing the configuration of the three‐electrode hybrid structure. An impressive solution, an all‐in‐one device combining piezo and triboelectric effects, has been reported, consisting of a top Ag electrode and a bottom Cu electrode utilizing a BTO ceramic sheet as the piezoelectric layer.^[^ ^22^ ^]^ The Ag electrode was placed on the BTO ceramic sheet, whereas the Cu electrode was positioned beneath it (Figure 6h). A unique feature of this device was the small hole drilled through the commercial PTFE film, serving as the triboelectric layer. The hole acted as a conductive channel that enabled the piezoelectric charges generated in the BTO layer to reach the bottom Cu electrode through the triboelectric PTFE layer, allowing both piezoelectric and triboelectric outputs to be collected through a shared external circuit. During operation, when the PTFE film contacted the Cu electrode, an electrostatic field was created, causing a flow of transferred triboelectric charges from the Ag electrode to the Cu electrode. The small conductive channel created by the hole in the PTFE film enabled efficient coupling and extraction of both piezoelectric and triboelectric signals through a common output pathway. This design enhanced the overall energy generation and conversion capability of the PT‐HNG during a single press‐and‐release cycle.

## 4 Key Factors of Performance Enhancement for the PT‐HNGs

### 4.1 Materials

In Hybrid Nanogenerators (HNGs), the choice of materials plays a pivotal role in achieving synergistic enhancement of energy conversion efficiency. The integration of triboelectric and piezoelectric functionalities demands careful consideration of material properties, as these directly impact charge transfer efficiency, mechanical durability, and energy harvesting capabilities.^[^ ^1^, ^2^, ^3^ ^]^ Understanding the interplay between materials for TENGs and PENGs is essential for optimizing hybrid designs. Utilizing nanofillers to form nanocomposite structures is an effective strategy to achieve elevated output from nanogenerators. 0D nanoparticles, such as KNN,^[^ ^89^ ^]^ BTO,^[^ ^90^ ^]^ quantum dots (Graphene, ZnO and PbS),^[^ ^91^, ^92^ ^]^ and metallic nanoparticles (Au, Ag, Sn, Ga and In);^[^ ^93^, ^94^, ^95^, ^96^ ^]^ 1D materials, including zinc oxide (ZnO) nanowires,^[^ ^97^, ^98^, ^99^ ^]^ aluminum nitride (AlN) nanowires^[^ ^100^, ^101^ ^]^ and PZT nanofibers,^[^ ^102^, ^103^, ^104^ ^]^ and 2D materials, including tin disulfide (SnS_2_),^[^ ^105^ ^]^ graphene,^[^ ^27^, ^106^, ^107^, ^108^ ^]^ MXene^[^ ^28^, ^109^, ^110^, ^111^ ^]^ and molybdenum disulfide (MoS_2_) nanosheets,^[^ ^112^, ^113^ ^]^ are considered some of the utilized fundamental nanofillers in nanogenerator applications. PVDF and its copolymers have strong electroactivity that can be utilized as active regions of nanogenerators.^[^ ^27^, ^28^, ^113^ ^]^ Furthermore, integrating low‐dimensional nanomaterials in PVDF enhances the piezoelectric and triboelectric effects, leading to fascinating performances in nanogenerator applications.^[^ ^29^, ^114^, ^115^, ^116^, ^117^, ^118^, ^119^ ^]^

In TENGs, several factors are crucial for achieving high output performance, among which is the selection of suitable material pairs. The triboelectric properties of polymers and other TENG materials, particularly the ability to gain or lose electrons during contact, are key determinants of performance. These properties can be systematically utilized to design and fabricate optimized TENG electrodes by leveraging the established triboelectric series. Standardized processes for designing high‐performance TENGs provide valuable guidelines for TENG development.^[^ ^120^ ^]^ However, more than mere selection of appropriate materials is required to guarantee optimal performance. In some instances, pure materials may exhibit suboptimal output, which can be addressed by incorporating nano‐ or micro‐materials (composites, fillers, and others), such as piezoelectric additives, to enhance the overall piezoelectric and dielectric properties of the flexible TENG. Furthermore, the type, morphology, and size of the chosen piezoelectric materials significantly influence TENG performance. Additionally, the size and thickness of the active materials, the operational mode, and environmental factors, such as temperature and humidity, must also be carefully considered, as they directly impact the efficiency and output of TENGs.

Furthermore, the morphology of piezoelectric nanofillers‐whether 0D nanoparticles, 1D nanorods/nanowires, or 2D nanofibers‐significantly influences charge generation, dipole alignment, and interfacial coupling in PT‑HNG systems. In electrospun PVDF‐ZnO nanofiber composites, the growth of ZnO nanorods on fiber surfaces enhances output performance by increasing surface roughness, boosting triboelectric charge, and improving piezoelectric coupling.^[^ ^121^ ^]^ In contrast, 0D nanoparticles (e.g., ZnO NPs) act primarily as β‑phase nucleating agents, improving dielectric properties, but lack the mechanical alignment benefits provided by 1D structures.^[^ ^122^ ^]^ These morphology‐dependent effects are applicable to both two‑electrode and three‑electrode configurations, influencing output magnitude, signal decoupling, and device impedance.

**Figure 7:**
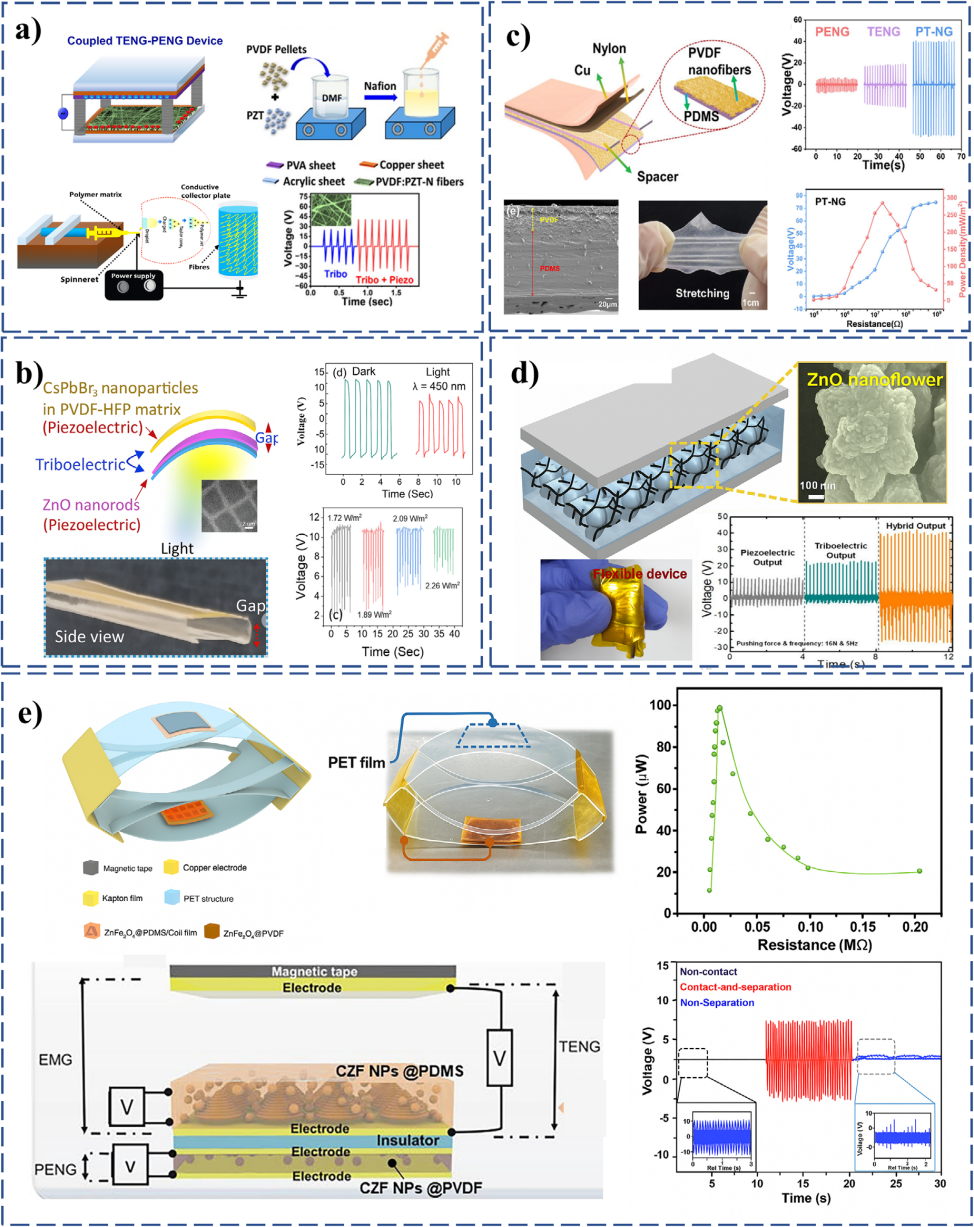
Influence of materials on enhancing the performance of PT‐HNGs. a) Schematic of a PVDF:PZT‐N PT‐HNGs device, showing an output voltage increase from 20.15 V to 40.2 V with PT‐HNG integration. Reprinted (Adapted) with permission.^[^ ^123^ ^]^ Copyright 2024, American Chemical Society. b) Hybrid ZnO‐CsPbBr_3_/PVDF‐HFP PT‐HNGs design, with HRTEM of CsPbBr_3_ nanoparticles and voltage variation under dark and visible light. Reprinted (Adapted) with permission.^[^ ^124^ ^]^ Copyright 2024, American Chemical Society. c) PVDF–PDMS composite film under stretching, SEM morphology, and comparisons of VOC and power density for PENG, TENG, and PT‐NG. Reprinted (Adapted) with permission.^[^ ^66^ ^]^ Copyright 2024, American Chemical Society. d) Hybrid MWCNTs/ZnO/PDMS PT‐HNG device, showing high flexibility and superior VOC output. Reprinted (Adapted) with permission.^[^ ^82^ ^]^ Copyright 2018, American Chemical Society. e) 3D schematic and photograph of PT‐HNG, with TENG and EMG electrodes, illustrating voltage output and power density performance across modes. Reprinted (Adapted) with permission.^[^ ^125^ ^]^ Copyright 2023, Elsevier.

Incorporating PZT, a piezoelectric and ferroelectric material, into polymer matrices was demonstrated to significantly improve the performance of PT‐HNGs.^[^ ^123^ ^]^ Their PVDF: PZT‐Nefion (PVDF: PZT‐N) composite exhibited a substantial enhancement in the *V_OC_* of up to 40 V and *I_SC_* of up to 7 µA output performances, as shown in **Figure** **7**a for *V_OC_* in comparison with only the triboelectric region. This improvement was attributed to the presentation of PZT and Nefion, which enhanced charge storage, polarization effects, and dipole alignment under mechanical stress within the matrix, making the composite highly effective for flexible and self‐powered electronics. Similarly, incorporating ZnO nanorods and CsPbBr_3_ nanoparticles into PT‐HNG was reported to significantly improve energy conversion efficiency and photodetection in the UV‐visible range (Figure 7b).^[^ ^124^ ^]^ ZnO nanorods, known for their strong piezoelectric properties, and CsPbBr_3_ nanoparticles with superior dielectric characteristics were integrated into PVDF‐HFP films to form PT‐HNGs. This PT‐HNG demonstrated an impressive output *V_OC_* of 28.2 V, an *I_SC_* of 29.6 nA, and photoresponsivity values of ≈6.59 × 10^4^ V/W in the visible region and ≈3.67 × 10^4^ V/W in the UV region. In detail, these enhancements were due to the synergistic interaction between triboelectric and piezoelectric effects, positioning ZnO‐CsPbBr_3_ systems as promising candidates for flexible, wearable energy and photodetection devices.

Developing PVDF‐PDMS composite film further advanced PT‐HNG designs synergistically enhanced the device's flexibility and energy‐harvesting capabilities.^[^ ^66^ ^]^ This composite leveraged the β phase of PVDF, known for its superior piezoelectric properties, while PDMS enhanced the triboelectric effect with its high electron affinity. The PT‐HNG achieved a *V_OC_* of 88 V and a power density of 286 mWm^−2^, surpassing the performance of conventional TENGs (136.72 mW m^−2^) and PENGs (40.53 mW m^−2^) under similar conditions Figure 7c. A PT‐HNG using a multiwalled carbon nanotube (MWCNT)/ZnO/PDMS composite film was also introduced.^[^ ^125^ ^]^ This design combined ZnO nanoflowers, known for their piezoelectric properties, with PDMS, which also enhanced surface roughness and further boosted triboelectric performance. Incorporating MWCNTs helped decrease the internal resistance of the PT‐HNGs and facilitated the distribution of ZnO nanoflowers. This device achieved an impressive *V_OC_* ​ of 150 V and a power density of 0.26 W cm^−2^, successfully lighting up 20 commercial LEDs connected in series. These results significantly improve over previous PT‐HNGs, making this device a strong candidate for energy harvesting from various human activities Figure 7d.

A PT‐HNG capable of multimodal mechanical energy harvesting was reported by embedding cuboctahedron ZnFe_2_O_4_ Nanoparticles in polymer nanocomposites.^[^ ^125^ ^]^ The device effectively operated under non‐separation, contact‐separation, and non‐contact modes, depending on the intensity of the applied mechanical force. Two nanocomposites were utilized: ZnFe_2_O_4_ Nanoparticles in a PDMS matrix, which facilitated TENG and electromagnetic generator (EMG) energy harvesting under vibrations and contact‐separation conditions, and ZnFe_2_O_4_ Nanoparticles in a PVDF matrix, which enhanced PENG energy harvesting by increasing the β phase fraction of PVDF, improving its response to mechanical pressure. The device achieved a rectified output *V_OC_* of 30 V and *I_SC_* of 3.5 µA, underscoring the effectiveness of combining triboelectric, piezoelectric, and additional electromagnetic mechanisms for improved energy harvesting performance Figure 7e.

### 4.2 Surface Modification

Surface modification plays a pivotal role in enhancing the performance of PT‐HNGs by improving critical factors such as surface area, piezoelectric properties, charge density, and overall stability, particularly during the contact‐separation processes, where increased surface roughness leads to enhanced energy generation.^[^ ^126^, ^127^, ^128^, ^129^ ^]^ Techniques such as plasma treatment, etching, surface chemical functionalization doping, fabrication of micro/nanostructured patterns, and others influence these parameters.^[^ ^128^, ^130^, ^131^ ^]^ In addition to enhancing the triboelectric properties, surface modification techniques can significantly influence the piezoelectric behavior of active materials. For instance, plasma treatment and surface nanopatterning can induce local stress fields and enhance dipole alignment within piezoelectric polymers like PVDF, increasing the proportion of the electroactive β‐phase, which is responsible for higher piezoelectric response.^[^ ^132^ ^]^ Similarly, doping with nanoparticles such as BaTiO_3_ or ZnO not only enhances interfacial polarization but also improves crystallinity and piezoelectric coefficient by facilitating stress‐induced dipole reorientation.^[^ ^133^, ^134^ ^]^ These modifications lead to improved strain sensitivity and charge generation in the piezoelectric component of PT‐HNGs.

Several studies have demonstrated that creating micro‐ and nano‐scale roughness on the surface of the active layers in TENGs and PT‐HNGs devices can substantially increase the contact area, thereby boosting the generated electrical output.

**Figure 8:**
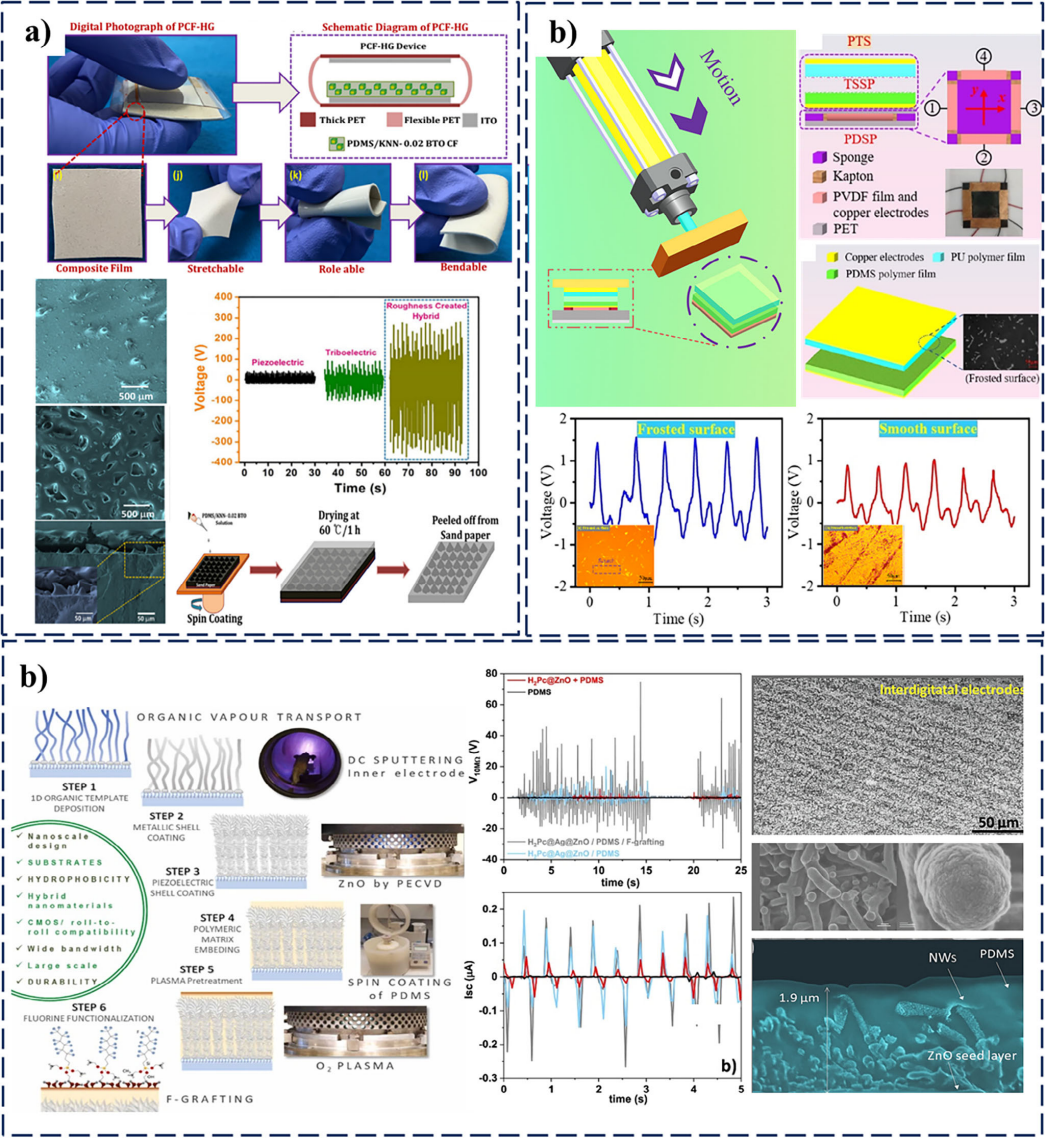
Influence of surface modification on enhancing the performance of PT‐HNGs a) Digital photo and schematic of the device, illustrating its flexible composite film and micro‐roughness created via soft‐lithographic sandpaper method. The comparison of VOC responses highlights enhanced output due to increased roughness. Reprinted (Adapted) with permission.^[^ ^135^ ^]^ Copyright2020, Elsevier. b) The working mechanism of the triboelectric state sensing part (TSSP) with SEM images of the forested surface shows transient voltage signals influenced by surface treatments. Reprinted (Adapted) with permission.^[^ ^136^ ^]^ Copyright 2023, Elsevier. c) Schematic of device fabrication and assembly, featuring O2 plasma‐treated interdigitated microelectrodes, core@multishell nanowires in a PDMS layer, and enhanced PT‐HNGs performance through architectural optimization. Reprinted (Adapted) with permission.^[^ ^137^ ^]^ Copyright 2022, Elsevier.

The impact of surface roughness on the performance of PT‐HNG, consisting of a composite film of (1‐x) K_0.5_Na_0.5_NbO_3‐x_ BaTiO_3_ nanoparticles embedded in a PDMS matrix (x = 0, 0.02, 0.04, 0.06, and 0.08), was investigated.^[^ ^135^ ^]^ This surface modification has shown that its performance as measured by the electrical output of the device was significantly higher when a sandpaper template was used to create a dense and uniform surface roughness on the composite film compared to a flat film (**Figure** **8**a). The roughened surface increased the number of contact points during the contact‐separation process, leading to a maximum electrical output of 610 V and 13.7 µA, which was two‐fold higher than the output from the flat film. Similarly, surface frosting and Nanoparticles doping in a PT‐HNG sensor was employed as surface modifications to enhance its performance.^[^ ^136^ ^]^ The surface frosting technique, in particular, improved the charge transfer efficiency, resulting in a 39.05% higher voltage output than smooth surfaces. This modification not only increased the friction coefficient but also reduced surface damage caused by impurities, further enhancing the durability and output of the sensor (Figure 8b).

Plasma‐assisted deposition techniques were utilized to modify the surface of PDMS layers in PT‐HNGs.^[^ ^137^ ^]^ The oxygen plasma treatment was employed to activate the PDMS surface by oxidizing the methyl‐siloxane groups (‐Si‐CH_3_), leading to the formation of silanol groups (‐Si‐OH) and silica‐like (SiO_x_) species. This treatment increases the surface energy and introduces reactive sites that facilitate subsequent chemical functionalization. Following plasma activation, perfluorinated silane molecules are grafted onto the modified PDMS surface through a silanization reaction, in which the hydrolyzed silane groups form covalent Si‐O‐Si bonds with the surface ‐OH groups. This process results in the formation of a stable, densely packed monolayer of perfluorinated chains. Introducing these fluorinated groups significantly enhances surface hydrophobicity and surface charge density, which are critical for boosting triboelectric output. Surface modification of the PDMS triboelectric active layer through plasma treatment, combined with the incorporation of Ag@ZnO nanowires (core@multishell) as piezoelectric components, made synergistic coupling between the triboelectric and piezoelectric mechanisms (Figure 8c). Operating in vertical contact‐separation mode, the resulting PT‐HNG efficiently harvested mechanical vibration energy from 1 Hz to 800 Hz over a broad frequency range. Moreover, the plasma‐engineered surface improved device durability and stability under humid conditions, ensuring reproducible and long‐term energy harvesting performance.

### 4.3 Device Design and Electronic Circuit Aid

**Figure 9:**
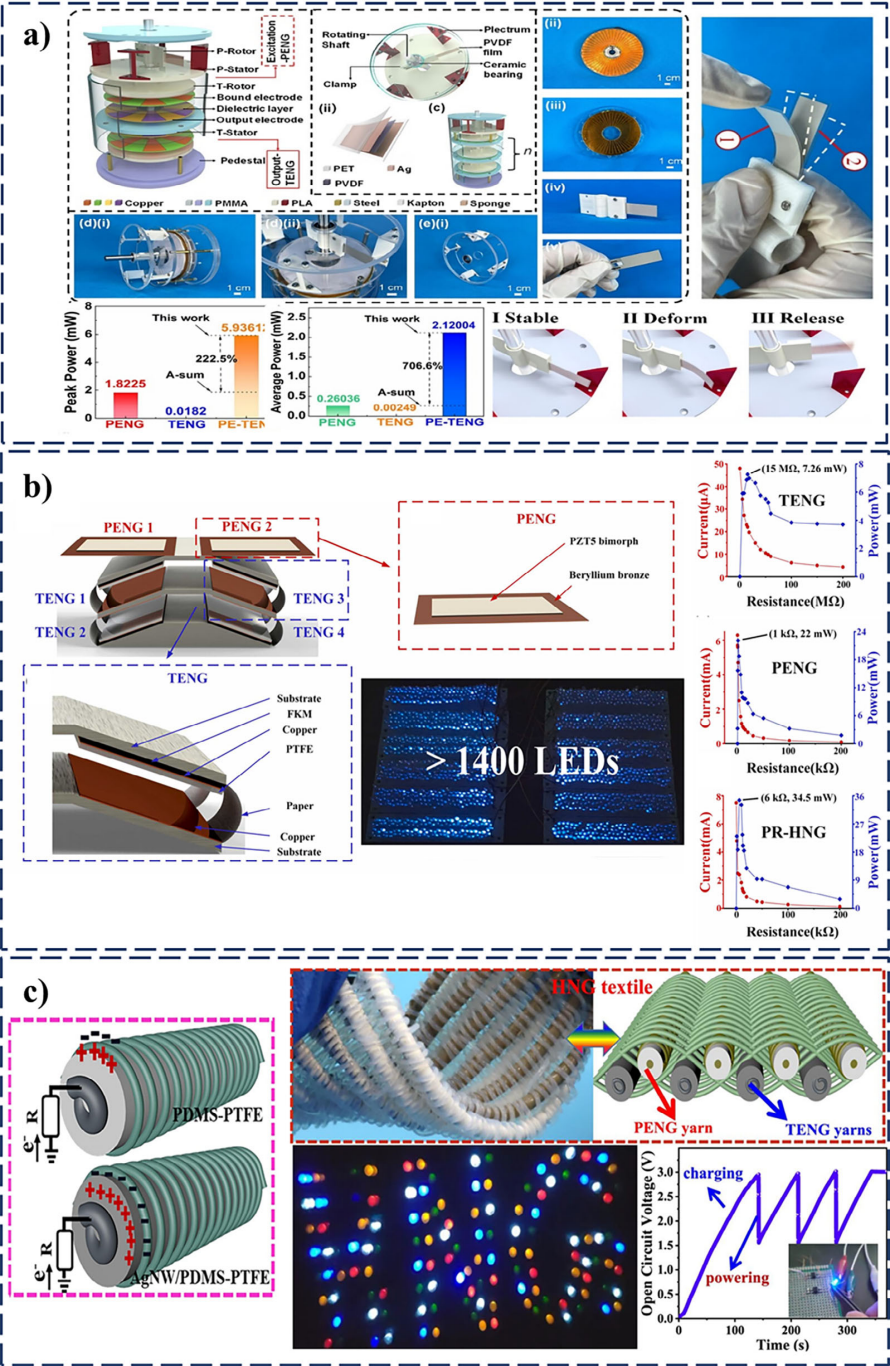
Influence of device design and electronic circuit aid on enhancing the performance of PT‐HNGs a) Schematic of PT‐HNG, showing exploded views of components, 3D excitation‐PENG structure, and PVDF film operation under rotational excitation, with a performance comparison of PENG, TENG, and PE‐TENG outputs. Reprinted (Adapted) with permission.^[^ ^139^ ^]^ Copyright 2023, Elsevier. b) Structural composition of PR‐PT‐HNG, comparing output power of TENG and PENG to the hybrid PR‐PT‐HNG, which powers over 1400 LEDs. Reprinted (Adapted) with permission.^[^ ^140^ ^]^ Copyright 2024, Elsevier. c) Structural composition of PR‐PT‐HNG, comparing output power of TENG and PENG to the hybrid PR‐PT‐HNG. Reprinted (Adapted) with permission.^[^ ^141^ ^]^ Copyright 2023, Elsevier.

Enhancing energy conversion efficiency in PT‐HNGs requires optimized device design and circuit integration. Recent advancements have led to notable improvements in these areas, supporting scalability for practical applications. A PT‐HNG was reported utilizing a charge‐pumping strategy.^[^ ^138^ ^]^ The system consists of two main components: the excitation‐PENG and the output‐TENG, both mounted on a shared rotating shaft. The PENG employs a flexible PVDF film positioned between insulating layers with bilateral electrodes, operating via the periodic deformation of the PVDF film during shaft rotation. The TENG features a complementary sector electrode design made of Cu layers on a printed circuit board, with a Kapton film serving as the insulator. Sponges were inserted between the disk base and electrodes to maintain consistent electrical contact during operation (**Figure** **9**a). This hierarchical design allows the PENG to generate instantaneous high‐energy outputs that are transferred to the TENG, increasing its surface charge density, which significantly enhances both peak and average power output. Finally, the PT‐HNGs demonstrated excellent performance, achieving a peak power of 5.9 mW and an average power of 2.1 mW at a drive frequency of 2.5 Hz. This corresponds to a 222.5% increase in peak power and a 706.6% increase in average power compared to the combined output power of the individual TENG and PENG devices.

A pitched roof‐like PT‐HNG was designed to harvest vibrational energy efficiently by integrating four TENG units and two PENG units within a flexible pitched roof structure (Figure 9b).^[^ ^140^ ^]^ This innovative PT‐HNG operates through the vibration‐induced opening and closing of its modules, generating electricity via both triboelectric and piezoelectric effects. The TENG units, consisting of PTFE, Cu, and fluoroelastomer rubber (Fluorine Kautschuk Material, FKM), produce charge separation through electron transfer, where PTFE acts as an electronegative material that readily gains electrons during contact with Cu. The PENG units, constructed from PZT‐5 bicrystal piezoelectric ceramics and conductive beryllium bronze alloy, harness energy through mechanical deformation. The inclusion of an electropositive FKM layer in the TENG units enhances charge induction, significantly improving energy output. Moreover, the flexible‐pitched roof structure minimizes mechanical wear, ensuring stable and long‐term performance. The device achieved a *V_OC_* of 800 V for the TENG unit and an *I_SC_* of 9.6 mA for the PENG unit. When combined, the PT‐HNG delivered a *V_OC_* and *I_SC_* of 800 V and 9.6 mA, respectively, with a maximum power output of 34.5 mW. This system could charge a 2000 µF capacitor to 2.5 V within 30 s. The stored energy was sufficient to power 1440 LEDs and small electronic devices, demonstrating the efficiency and practicality of the PT‐HNG's intelligent design and hybrid configuration.

A highly efficient PT‐HNG was developed by incorporating a 3D trinary‐yarn‐interlocked parallel‐arranged structure to maximize the contact area for enhanced energy conversion.^[^ ^141^ ^]^ The textile comprises three types of elastomer yarns: piezoelectric BTO/PDMS yarns with a Cu wire core as the inner electrode and a coil spring outer electrode, which also serves as the triboelectric electrode. Ag nanowire/PDMS elastomer yarns, coated on a coil spring, share a similar structure and work with PTFE yarns. The PTFE yarn was chosen for its strong electron‐accepting properties due to carbon‐fluorine bonds, further enhancing the triboelectric interactions. The PTFE yarn is knitted around BaTiO_3_/PDMS and Ag nanowire/PDMS yarns in an alternating up‐and‐down parallel arrangement, forming a 3D trinary‐yarn‐interlocked configuration that maximizes energy generation during mechanical deformations such as stretching, bending, or twisting (Figure 9c). This design allows the outer coil spring electrode to serve as both the secondary piezoelectric and primary triboelectric electrodes, ensuring effective coupling between the two effects. Operating in double‐electrode enhancement mode, the device achieves a peak power density of 91.6 mWm^−^^2^ and a maximum output *V_OC_* of 400 V. Compared to conventional 2D or planar hybrid nanogenerators, this textile design demonstrates significantly improved energy output, owing to the optimized 3D interlocked structure and the use of materials with distinct electron‐accepting and ‐donating properties.

## 5 Piezoelectric–Triboelectric Hybrid Nanogenerator Output Enhancement Considerations

Building on the principles discussed in Section 4, this section elaborates on material‐level surface modification techniques employed to improve the performance of PT‐HNGs, which integrate triboelectric and piezoelectric mechanisms through a unified structural design, and offer enhanced energy conversion efficiency. By optimizing the triboelectric and piezoelectric components, the total output of PT‐HNGs can be significantly improved, leveraging advancements from both fields to achieve higher output and performance.

### 5.1 Tribo‐enhanced PENGs

In the case of TENGs, their output performance can be considerably enhanced through the careful selection and design of specific parameters. In a typical contact‐separation mode, both the *V_OC_* and *I_SC_* are influenced by independent factors. These relationships are described by the following equations:^[^ ^142^ ^]^

(9) $$V_{OC}=\frac{\sigma \;x}{\varepsilon_{0}}\;$$

(10) $$I_{SC}=\frac{S\sigma x}{d_{0}+x}\;$$

where σ is the triboelectric charge density, *x* is the separation distance between the triboelectric layer and the movable electrode, ε_0_ is the dielectric constant of the triboelectric layer, *d*_0_ is the thickness of the triboelectric layer, and *S* is the contact area between the triboelectric layer and the electrode. *The* *x* can be changed in the range of 0 to a certain value, but for a given triboelectric material, σ,  ε_0_, *d*_0_ remain constant. So, these analyses show that the contact area, *S*, can positively change, and increasing the contact area leads to a direct improvement in the triboelectric effect, which is essential for optimizing energy harvesting efficiency in PT‐HNGs. Various surface modification techniques increase surface roughness and enhance contact intimacy, improving charge transfer and overall device performance.

**Figure 10:**
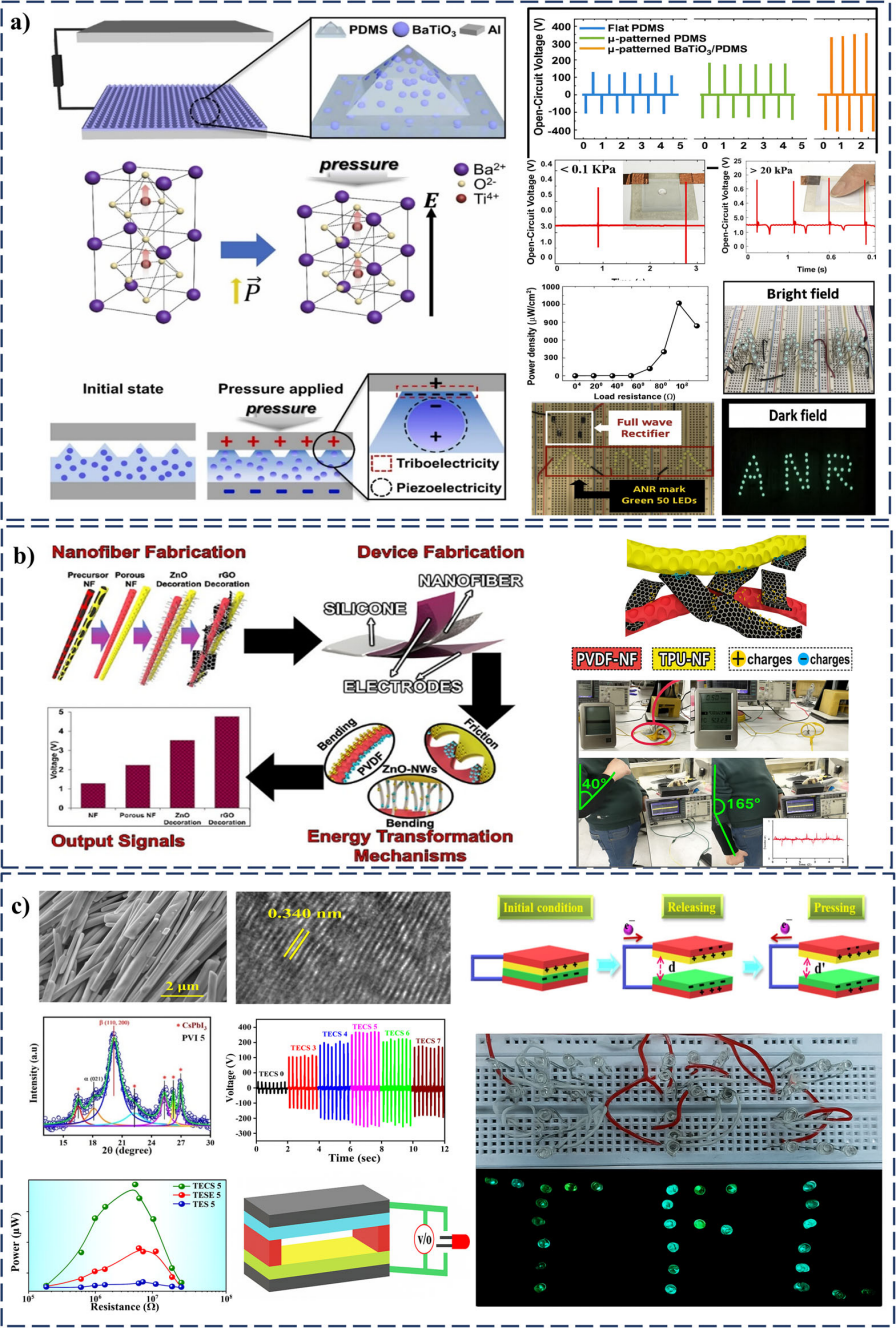
TENG enhances PENG for enhanced PT‐HNGs output a) Schematic of the micro‐pyramidal BaTiO_3_/PDMS TENG, BaTiO_3_ crystal structure before and after pressure, and V_OC_ comparison of different PDMS‐based TENGs. Real‐time V_OC_ responses, power density vs. load resistance, circuit diagram, and LED demonstration. Reprinted (Adapted) with permission.^[^ ^143^ ^]^ Copyright 2024, Elsevier. b) Fabrication and surface modification of NFs, PT‐HNG energy conversion mechanism, charge transfer via graphene flakes, textile sensor testing, and SEM images of roughened NFs. Reprinted (Adapted) with permission.^[^ ^144^ ^]^ Copyright 2023, American Chemical Society. c) FESEM and HRTEM images of CsPbI_3_, with the deconvoluted X‐ray diffraction (XRD) profile of PVI 5, confirm the high crystallinity. Schematic diagrams depict the working mechanism of the device and its electrical performance, including V_OC_, which is shown for all devices, with TENG power output in different modes. Additionally, including a digital image of the humidity sensing circuit setup, schematic design of the self‐powered humidity sensor in triboelectric contact‐separation mode (HTECS), and the glowing LEDs in the ‘TFL’ pattern by HTECS Reprinted (Adapted) with permission.^[^ ^62^ ^]^ Copyright 2024, American Chemical Society.

Triboelectric performance was enhanced by fabricating a micro‐pyramidal pattern on the PDMS surface and incorporating BaTiO_3_ nanoparticles through micropatterning (**Figure** **10**a).^[^ ^143^ ^]^ Specifically, the micro‐pyramidal structures notably increased the triboelectric contact area and charge density, while the addition of BaTiO_3_ nanoparticles further enhanced both piezoelectric and triboelectric charge generation. Under applied pressure, the hybrid BaTiO_3_/PDMS PT‐HNG produced an output *V*_*OC*_ of 372.4 V, an *I_SC_* of 81.3 µA, and a charge density of 91.8 µC m^‐^^2^. Furthermore, the device achieved a power density of ≈1.5 mW cm^‐^^2^, underscoring its potential for advanced energy harvesting and high‐sensitivity pressure sensing applications over a wide pressure range (0.1 kPa to 100 kPa).

An innovative 3D PT‐HNG was developed by integrating surface roughening techniques with nanomaterial decorations as triboelectric enhanced properties.^[^ ^144^ ^]^ The PT‐HNGs consist of hybrid nanofibers made from a blend of PVDF and TPU mixed with Polyvinylpyrrolidone (PVP). A chemical etching process was employed to remove PVP, generating a porous structure that enhanced surface roughness and improved triboelectric charge generation (Figure 10b). These modifications significantly improved the contact area and charge transfer efficiency, leading to a 75% increase in voltage and a 169.23% increase in current compared to neat PT‐HNGs. The roughened NFs were decorated with vertically aligned ZnO nanowires through hydrothermal growth, and reduced oxide (rGO) nanoparticles were electrosprayed onto the surface to optimize performance further. The vertically aligned ZnO nanowires notably enhanced the piezoelectric response, boosting energy generation during mechanical deformation. At the same time, the rGO Nanoparticles reduced internal resistance, facilitating higher charge transfer and increasing effective surface area by enhancing triboelectric properties. The combination of ZnO nanowires and rGO Nanoparticles yielded remarkable improvements in performance, with a 271.80% increase in voltage density and a 230.77% increase in current density, achieving peak values of 2.35 kV m^−2^ and 3.40 mA m^−2^, respectively.

On the other hand, enhancing the triboelectric charge density is a promising strategy to improve the output performance of PT‐HNGs. This can be achieved by selecting triboelectric materials with greater position differences within the triboelectric series or other modifications. For instance, CsPbI_3_ was incorporated into a PVDF matrix to increase the electroactive phase and dielectric permittivity, thereby enhancing surface charge density.^[^ ^62^ ^]^ The CsPbI_3_–PVDF composite film, used in both piezoelectric and triboelectric layers, demonstrated superior performance across multiple modes, including triboelectric contact‐separation (TECS), single‐electrode, and sliding modes, achieving power densities of 3.08 mW, 1.29 mW, and 0.15 mW at an external load of 5.6 MΩ, respectively (Figure 10c). The high crystallinity and strong dielectric properties of PVI 5 (PVDF with 5 wt% of CsPbI_3_) in TECS 5 contributed significantly to increasing triboelectric charge density, as more charges accumulated on the negatively charged PVI 5 film and the positively charged nylon surface, which have opposite polarities. This example underscores how triboelectric material modifications can substantially enhance energy harvesting efficiency in PT‐HNGs, making them strong candidates for applications such as wearable health monitoring, biometric security systems, and other emerging technologies.

### 5.2 Piezo‐Enhanced TENGs

**Figure 11:**
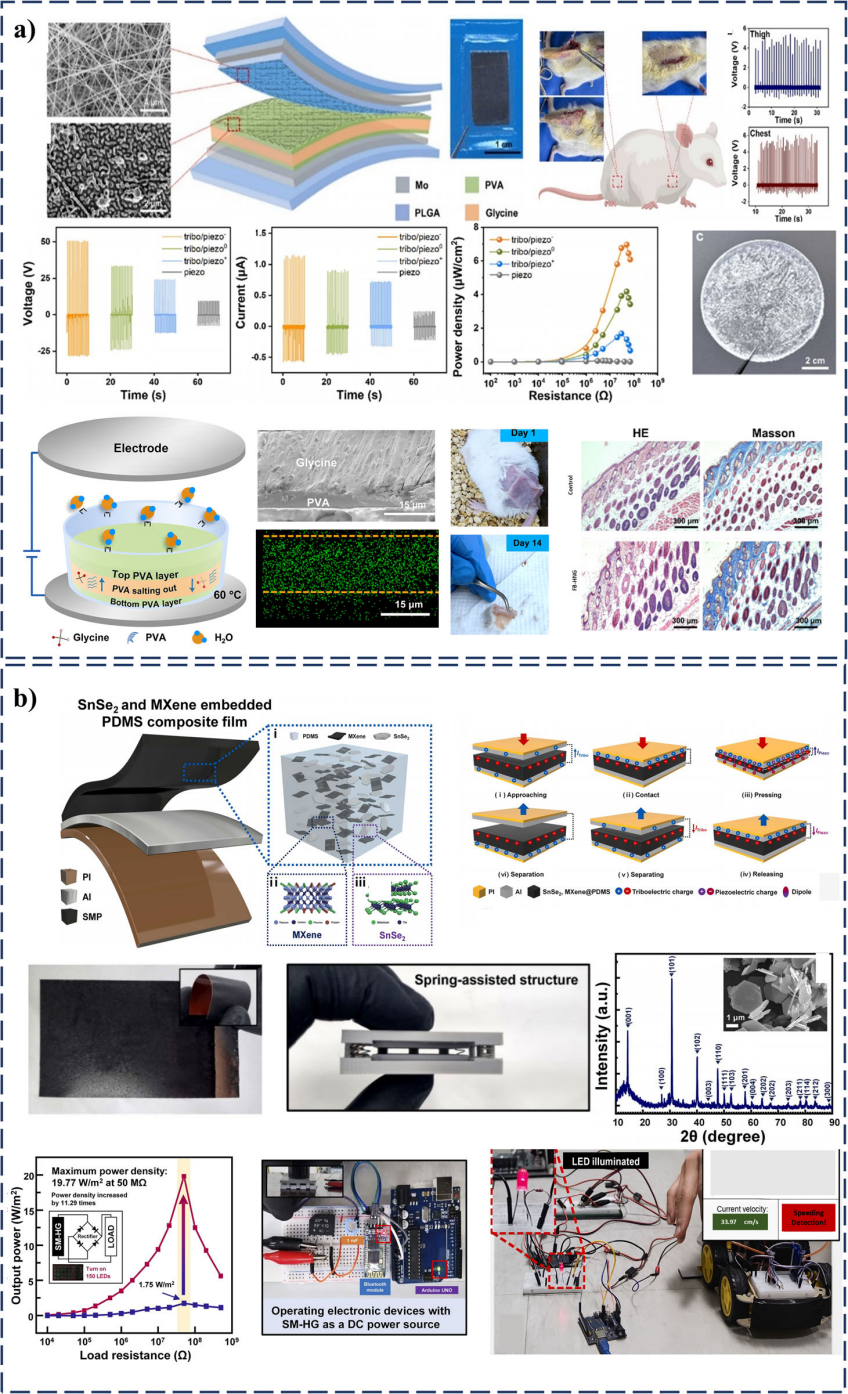
PENG enhances TENG for enhanced PT‐HNGs output. a) Schematic of the PT‐HNG, SEM images of PLGA nanofibers and PVA/Gy/PVA film, and output comparisons under different coupling conditions. In vivo analysis includes implantation images, toxicity assessment, and electrode degradation over 14 days. Wafer‐scale film preparation with SEM/EDS mapping is shown. Reprinted (Adapted) with permission.^[^ ^145^ ^]^ Copyright 2024, Elsevier. b) PT‐HNG structure with SnSe_2_‐MXene‐embedded PDMS, XRD/SEM characterization, and enhanced output in vertical contact‐separation mode. Power density comparison, capacitor charging, and application in a speed detection system are demonstrated. Reprinted (Adapted) with permission.^[^ ^146^ ^]^ Copyright 2024, Elsevier.

Piezoelectric performance can be enhanced by incorporating piezoelectric nanomaterials into the design of PT‐HNGs. This addition increases the piezoelectric constant of the piezoelectric component, ultimately improving its overall properties. For example, high‐performance and fully biodegradable PT‐HNG for medical device applications was reported utilizing a sandwich‐like PVA/Glycine/PVA structure, where the glycine bulk layer acts as a piezoelectric material, significantly enhancing the device's output performance (**Figure** **11**a).^[^ ^145^ ^]^ A key innovation in this study is the application of an electric field‐assisted water evaporation technique, which improves the alignment of glycine dipoles and facilitates the formation of high‐quality crystals, resulting in a peak piezoelectric coefficient (d33) of 9 pC/N. The fully biodegradable PT‐HNG achieved an impressive maximum output *V_OC_* of 94 V, an *I_SC_* of 2.3 µA, a current density of 1.53 µA cm^‐^^2^, and a charge density of 6.53 nC cm^‐^^2^, representing the highest performance among biodegradable nanogenerators. Additionally, in vivo testing demonstrated stable electrical output exceeding 4 V upon implantation, alongside excellent biocompatibility and significant biodegradation within 14 days. In the other study, SnSe_2_/MXene@PDMS‐based PT‐HNG was developed to enhance the performance by integrating SnSe_2_, a piezoelectric and n‐type semiconductor, into a PDMS matrix, with MXene serving as a conductive filler.^[^ ^146^ ^]^ The incorporation of SnSe_2_ nanoparticles improved charge separation and storage under mechanical pressure (Figure 11b), generating additional piezoelectric charges. In detail, the electron‐donating properties of SnSe_2_, combined with the electronegativity of Sn and Se, created electron‐trapping sites that increased the dielectric constant, work function, charge density, and contact electrification. MXene contributed to a conductive network and micro‐capacitor system, significantly improving charge transfer, electron mobility, and dielectric properties within the PDMS. Additionally, including LiF during fabrication further enhanced the work function due to the high electron affinity of F. As a result, the device achieved a *V_OC_* of 202.31 V and an *I_SC_* of 108.55 µA, with a maximum power density of 19.77 W m^‐^^2^ at a load resistance of 50 MΩ. These characteristics highlight the potential of this design for flexible and wearable electronics, as well as for self‐powered sensors and energy‐efficient systems, including those used for detecting speeding vehicles.

## 6 Application of Piezo‐Triboelectric Hybrid Nanogenerators

Wearable hybrid nanogenerators are versatile tools with widespread applications in energy harvesting, self‐powered sensing, and bioelectronics. They provide real‐time data for sports analytics, safety alerts, radiation protection, environmental monitoring, and health diagnostics, demonstrating their diverse impact across healthcare, industrial safety, and environmental monitoring sectors.

### 6.1 Energy Harvesting

The primary focus of the research on PT‐HNGs is their use in energy harvesting from environmentally abundant mechanical sources like water waves, wind, raindrops, and vibrations.^[^ ^129^, ^130^, ^131^, ^132^, ^147^, ^148^, ^149^ ^]^ However, due to the limitation of low power output, PT‐HNGs have been employed to harvest mechanical energy at the microscale, which is used primarily on portable small devices rather than instruments that require large power input to run.^[^ ^150^, ^151^, ^152^ ^]^ Flexible wearable devices are made of functional composite films or fibers embedded with nanomaterials such as ZnO, BTO, and KNN and coupled with flexible materials acting as electrodes.^[^ ^83^, ^153^, ^154^ ^]^ PT‐HNGs are integrated with human wearables, including shoe insoles, hand gloves, and socks, or attached as patches to the elbow, torso, or extremities to capture human mechanical motion and run the wearable electronics devices.^[^ ^153^, ^155^ ^]^ Wearable PT‐HNGs, with the current technology, can produce sufficient energy to power small portable sensors.^[^ ^156^ ^]^

**Figure 12:**
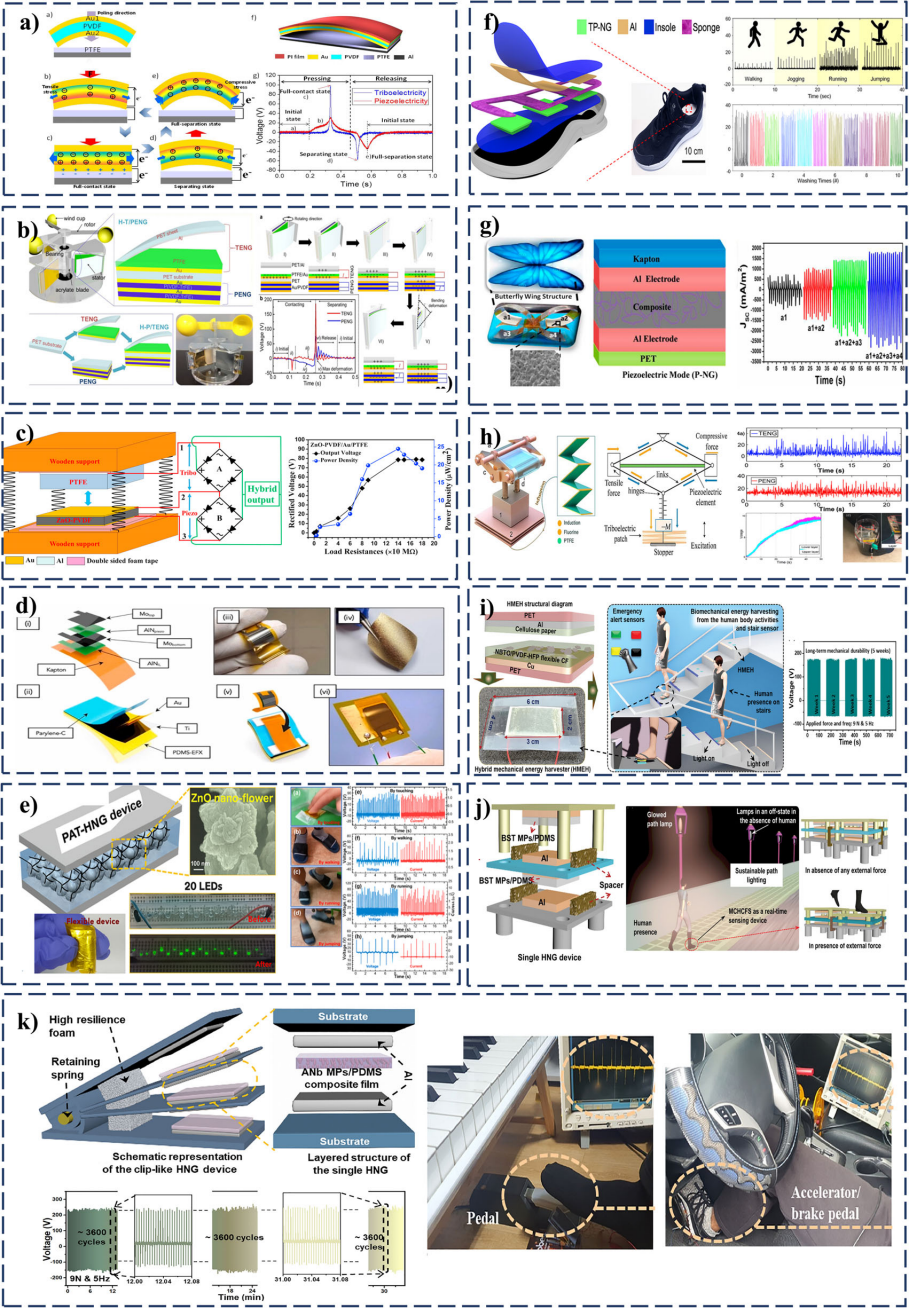
PT‐HNGs for energy harvesting applications, a) Working mechanism in a press‐and‐release cycle, and output performances. Reproduced (Adapted) with permission.^[^ ^79^ ^]^ Copyright 2015, Springer Nature. b) Schematic and working principle of a hybrid piezoelectric‐triboelectric nanogenerator (H‐P/TENG) for rotary energy harvesting. The inset shows the layered structure of the hybrid device including the fabrication process, working mechanism under deformation, and synchronous voltage outputs from the TENG and PENG components. Reprinted (adapted) with permission.^[^ ^60^ ^]^ Copyright 2019, Elsevier. c) PT‐HNG along with the electrical connections. Reprinted (Adapted) with permission.^[^ ^78^ ^]^ Copyright 2018, Elsevier. d) 3D expanded views of the PENG (i) and TENG (ii) components, with real photos (iii, iv); (v) assembly and (vi) real photo of the full PT‐HNG attached on a substrate. Reprinted (Adapted) with permission.^[^ ^160^ ^]^ Copyright 2021, Elsevier. e) Schematic diagram of flexible PT‐HNG via Polydimethylsiloxane‐Encapsulated Nanoflower‐like ZnO Composite Films. Reprinted (Adapted) with permission.^[^ ^82^ ^]^ Copyright 2018, American Chemical Society. f) Diagram and a digital photograph of PT‐HNG embedded in a shoe insole, rectified short‐circuit currents for various human motions. Reprinted (Adapted) with permission.^[^ ^86^ ^]^ Copyright 2020, Elsevier. g) Butterfly wing structure composed of four arc‐shaped HNG with an irregular surface morphology (a1, a2, a3, and a4); and output current density of single (a1) and multiunit HNGs connected in parallel at a constant acceleration. Reprinted (Adapted) with permission.^[^ ^161^ ^]^ Copyright 2018, Elsevier. h) Structural configuration of the PT‐HNG. Key components are listed as: 1. triboelectric patch, 2. proof mass, 3. linear spring, 4. truss mechanism, 5. piezoelectric plate and 6. piezoelectric plate holders. Reprinted (Adapted) with permission.^[^ ^163^ ^]^ Copyright 2018, Elsevier. i) The Components of the device include Biomechanical energy harvesting from human activities, smart home stair sensor, and emergency e‐mail alert applications. Reprinted (Adapted) with permission.^[^ ^164^ ^]^ Copyright 2024, John Wiley and Sons. j) Schematic diagram representing the reactions of MCHCFS in h) absence and i) presence of external force applied while human walking. Reprinted (Adapted) with permission.^[^ ^165^ ^]^ Copyright 2023, John Wiley and Sons. k) The clip‐like PT‐HNG device when placed on the pedal attached to the musical piano and the car's accelerator pedal with the working cycle. Reprinted (adapted) with permission.^[^ ^166^ ^]^ Copyright 2024, Elsevier.

An arch‐shaped hybrid structure was proposed with two vertically stacked layers, with the top piezoelectric layer made of Au/PVDF/Au and the bottom layer a triboelectric generator using a PTFE/Al structure (**Figure** **12**a).^[^ ^79^ ^]^ The structure enhances the output by integrating both mechanisms, resulting in a peak output voltage of 370 V, a current density of 12 mA cm^−^^2^, and an average power density of 4.44 mW cm^−2^. This PT‐HNG can light up 600 LED bulbs with a mechanical force of 0.2 N and under the maximum force, the generator‐powered 880 LED bulbs. A flapping‐leaf generator has been developed featuring two triangle‐shaped flapping leaves attached perpendicularly to the free ends of cantilevers, amplifying vibration motion for effective energy harvesting.^[^ ^157^ ^]^ The flapping base consists of a center frame sandwiched between metal films and PDMS layers, with flexible PVDF cantilevers coated in Al film symmetrically assembled on top and bottom. Pyramid microstructures on the PDMS film increase the contact area, enhancing voltage output. While evaluating the PT‐HNG with an industrial fan at wind speeds of 4.5 m s‐1, the piezoelectric part achieved a maximum power output of 8.1 µW with optimized load resistances of 1 MΩ. In contrast, the triboelectric part reached 13.6 µW, with optimized load resistances of 0.75 MΩ. A fully enclosed PT‐HNG has been developed with a double‐plate design, incorporating a nanotextured surface and piezoelectric fibers to effectively enhance the ability to harvest mechanical energy.^[^ ^158^ ^]^ This hybrid device demonstrated a peak output of 130 V and 4 µA at 2 Hz, resulting in an area power density of 8.34 mW m^−2^. By using a DC motor, the PT‐HNG was subjected to cyclic stretching at a strain of 0.5% and a frequency of 2 Hz. Different structural configurations exhibited output voltages of 40 V for the planar design, 58 V for irregular protrusions, and 77 V for the triangular surface. This device was also evaluated by harvesting energy from basketball dribbling at heights of 20 cm and 30 cm. The output voltage was 40 V and 72 V, with corresponding currents of 2.5 µA and 3.5 µA. The maximum area power density was 4.04 mW m^−^^2^ at 30 cm. A bimorph structure was developed with two PVDF‐TrFE films connected in parallel (Figure 12b), ensuring the output signals from TENG and PENG are in the same phase with the addition of the nanodots on the PTFE surface, increasing the active points for effective triboelectrification.^[^ ^60^ ^]^ At 14 m s^−1^ of wind speed, the PT‐HNG produced an output voltage of ≈150 V and a current of 150 µA, successfully powering 50 LEDs connected in parallel. The average output power of the PT‐HNG was estimated to be 10.88 mW with a matching impedance of ≈1 MΩ. A flexible PT‐HNG was developed using an electrospinning system for a three‐layer structure with piezoelectric PVDF‐TrFE nanofibers between wave‐shaped Kapton films, generating both piezoelectric and triboelectric outputs in one press‐release cycle.^[^ ^159^ ^]^ The upper TENG, made of PET‐ITO and Cu‐Kapton films, enhances electron transfer and exploits the combined effects of piezoelectric and triboelectric potentials, achieving a peak output of 96 V, surpassing the individual modes (47.8 V for piezoelectric and 64 V for triboelectric). Experimental tests confirmed the superior performance of PT‐HNG, with significant improvements in both voltage and current outputs. A ZnO‐PVDF composite‐based PT‐HNG was developed paired with PTFE featuring Al and Au layers for electrical contacts, enabling simultaneous energy harvesting (Figure 12c).^[^ ^78^ ^]^ Incorporating ZnO significantly boosts piezoelectric and triboelectric properties, increasing the piezoelectric coefficient by 23%, and raising output voltages, current density, and a 2.5‐fold increase in power output. Another flexible and ultrathin design (<100 µm) was reported that integrates piezoelectric and triboelectric mechanisms using biocompatible materials, including AlN, PDMS, and Ecoflex™ (Figure 12d).^[^ ^160^ ^]^ This integration enhanced energy harvesting from various water sources, i.e., raindrops and waves, achieving power densities of 6.5 mW m^−^^2^ for PENG, 65 mW m^−^^2^ for TENG, and 230 mW m^−^^2^ for the hybrid system. Experiments, including raindrop impact testing and wearable sensor demonstrations, showed the effectiveness of the device, with output energy reaching up to 0.8 W m^‐^^2^ under substantial impacts. This further validates its improved performance and multifunctionality. A piezoelectric‐assisted PT‐HNG was reported using a composite film of MWCNT, ZnO, and PDMS sandwiched between two Al substrates, with the bottom substrate acting as the electrode (Figure 12e).^[^ ^82^ ^]^ The key experimental procedures included optimizing the ZnO nanoflower concentration, with 4.8 wt% ZnO and 0.015 g of MWCNT producing the best electrical output, showing ∼75 V / 3.2 µA while walking, ∼150 V / 8 µA while running, and ∼400 V / 30 µA while jumping. A simple PT‐HNG was reported in which PVDF, Al electrodes, and acrylic supports were stacked vertically to harvest energy from foot vibrations (Figure 12f).^[^ ^86^ ^]^ Polarization control of the PVDF films enhances power output, with an up‐and‐down configuration producing 127 µW, compared to 52 µW for the down‐up configuration. The in‐phase power generation from the device's triboelectric and piezoelectric components can effectively capture random mechanical vibrations. Compressive forces simulated foot movements showed a maximum power output of 127 µW, which is sufficient to power LEDs and a wireless pressure sensor network. A multiunit system was developed inspired by butterfly wings (Figure 12g), integrating four arc‐shaped nanogenerators for dual functionality as an LED power source and a self‐powered air pressure sensor.^[^ ^161^ ^]^ Its irregular surface morphology, combined with piezoelectric nanoparticles (0.3BCT−0.7BST) in a PDMS matrix, significantly enhances surface charge density and output performance. Experimental results showed an open‐circuit voltage of 572 V and a power density of 1336 W m^‐^^2^, with the irregular surface outperforming flat and micropillar surfaces. These design improvements demonstrate HNG's potential for efficient energy harvesting applications. An advanced PT‐HNG was developed to effectively harvest energy from ocean waves.^[^ ^15^ ^]^ The grid‐like configuration enhanced parallel connections and delivered robust output performance in simulated wave conditions. The device exhibited a linear current, voltage, and charge transfer rise as wave frequency and amplitude increased. A significant voltage accumulation was observed in the capacitor charging test while the devices generated a maximum output of 140.93 µA and 229.31 V, with power reaching up to 19.76 mW. In a large‐scale setup with 11250 units, the estimated average output power was 21.61 W, demonstrating the device's potential for large‐scale energy harvesting from ocean waves. A PT‐HNG designed to optimize energy harvesting from ambient vibrations incorporates a hinged‐hinged PZT bimorph equipped with T‐shaped copper‐proof masses to reduce stress. The setup was encapsulated in a PMMA cube package, which included an adjustable bolt mechanism for fine‐tuning the resonant frequency.^[^ ^162^ ^]^ The TENG functioned as an overload protection stopper and a generator, while the PENG harvests vibration energy. Optimized for better linearity and sensitivity, the TENG could sense up to 1.5 g with 15 V g^−1^ sensitivity. The PENG achieved a 6.5 mW root mean square (rms) power output at 25 Hz and could light up 30 serial LEDs during vibrations. This design was assessed in a virtual reality train monitoring system, showing its potential for real‐world wireless sensor networks in harsh environments. Another PT‐HNG study reported that using a spring‐mass system with an amplitude limiter enhanced the conversion of mechanical vibrations into electricity.^[^ ^163^ ^]^ The triboelectric part used a zigzag‐shaped polyimide film to increase surface area for the charge generation, along with Cu electrodes and materials like Nylon and PTFE. PZT‐5J plate was used as the piezoelectric part with silver‐coated electrodes to maximize voltage output (Figure 12h). When assessed with vibration, the device exhibited 58.4 V from the piezoelectric part and 60 V from the triboelectric part, with a total power output of 19.6 mW. The device also exhibited rapid capacitance, reaching 7.6 V in 50 s. The amplitude limiter ensured the device resonated at 3 Hz, making it even better at collecting energy from low‐frequency movements. A high‐performance hybrid mechanical energy harvester was developed using niobium‐doped bismuth titanate (NBTO) embedded in a PVDF‐HFP matrix. The NBTO plates were synthesized via a molten‐salt technique and uniformly dispersed into the PVDF‐HFP solution to fabricate a flexible composite film with enhanced β‐phase fraction, piezo/ferroelectric properties, and dielectric performance.^[^ ^164^ ^]^ The fabricated device exhibited a stable electrical output of ≈175 V, 5.8 µA, and a surface charge density of 76 µC m^‐^^2^, with a maximum power density of 2.02 W m^−2^. This energy output was effectively utilized in practical applications such as innovative stair‐sensing systems, where the harvester was integrated with a microcontroller to trigger emergency alerts via a Wi‐Fi module, demonstrating its capability for real‐time monitoring and self‐powered IoT applications. The device maintained its performance over extended tapping cycles (>10000 cycles) and several days of operation, confirming its mechanical robustness and durability (Figure 12i). Another study reported strontium‐doped barium titanate microparticles embedded in a PDMS matrix to enhance dielectric and piezoelectric characteristics.^[^ ^165^ ^]^ The BST particles were synthesized via a solid‐state reaction and incorporated into PDMS to form a flexible composite triboelectric layer. Operating in contact‐separation mode against an aluminum electrode, the optimized device achieved a high electrical output of ≈280 V, 8.5 µA, and a surface charge density of 90 µC m^−2^. The nanogenerator underwent durability and robustness testing to validate long‐term performance, confirming stable output over repeated tapping cycles and varying force/frequency conditions. Eight such devices were integrated into a 3D‐printed flooring system designed to harvest mechanical energy from human footsteps for practical deployment (Figure 12j). The harvested energy was successfully utilized for sustainable path lighting, demonstrating the feasibility of this platform for real‐world energy harvesting from biomechanical motion. A hybrid mechanical energy harvester was developed by embedding lead‐free silver niobate (AgNbO_3_) microparticles into a PDMS matrix to form a composite film.^[^ ^166^ ^]^ The AgNbO_3_ microparticles were synthesized and uniformly dispersed into the PDMS polymer, which served as the negative triboelectric layer. The device was assembled in a clip‐like 3D‐printed structure (Figure 12k), where the PDMS/AgNbO_3_ composite film faced an aluminum electrode, forming a contact‐separation triboelectric configuration. Upon mechanical contact and separation‐such as pressing or vibration, the triboelectric effect between PDMS and aluminum, along with the piezoelectric response of AgNbO_3_, synergistically generated electrical output. The optimized device, containing 5 wt% AgNbO_3_, produced a high output of ≈340 V and 20 µA, sufficient to power small electronics and charge capacitors. The device's electrical performance was evaluated under various mechanical activations to assess reliability. It showed consistent output stability, confirming its mechanical robustness and durability (>10000 cycles) for repeated biomechanical energy harvesting scenarios, such as from a car accelerator pedal or piano foot pedal.

### 6.2 Self‐Powered Sensing

#### 6.2.1 Human Motion Monitoring

As a part of self‐powered sensing, human motion monitoring has widespread effects across various fields, including sports, healthcare, and rehabilitation, and is regarded as one of the fundamental applications of HNGs. By real‐time tracking of motion patterns, gait, and posture, wearable hybrid sensors can detect potential issues early, enabling corrective actions. Moreover, these sensors can enhance recovery output by continuously monitoring progress and ensuring adherence to prescribed therapy for people undergoing rehabilitation. Furthermore, PT‐HNGs are hugely beneficial to athletes for optimizing training strategies, improving performance metrics, and minimizing the risk of overuse injuries. Several wearable hybrid sensors have been employed in human motion monitoring, including elbow, wrist, and hand movement and gait analysis.^[^ ^82^, ^167^, ^168^, ^169^, ^170^, ^171^ ^]^

**Figure 13:**
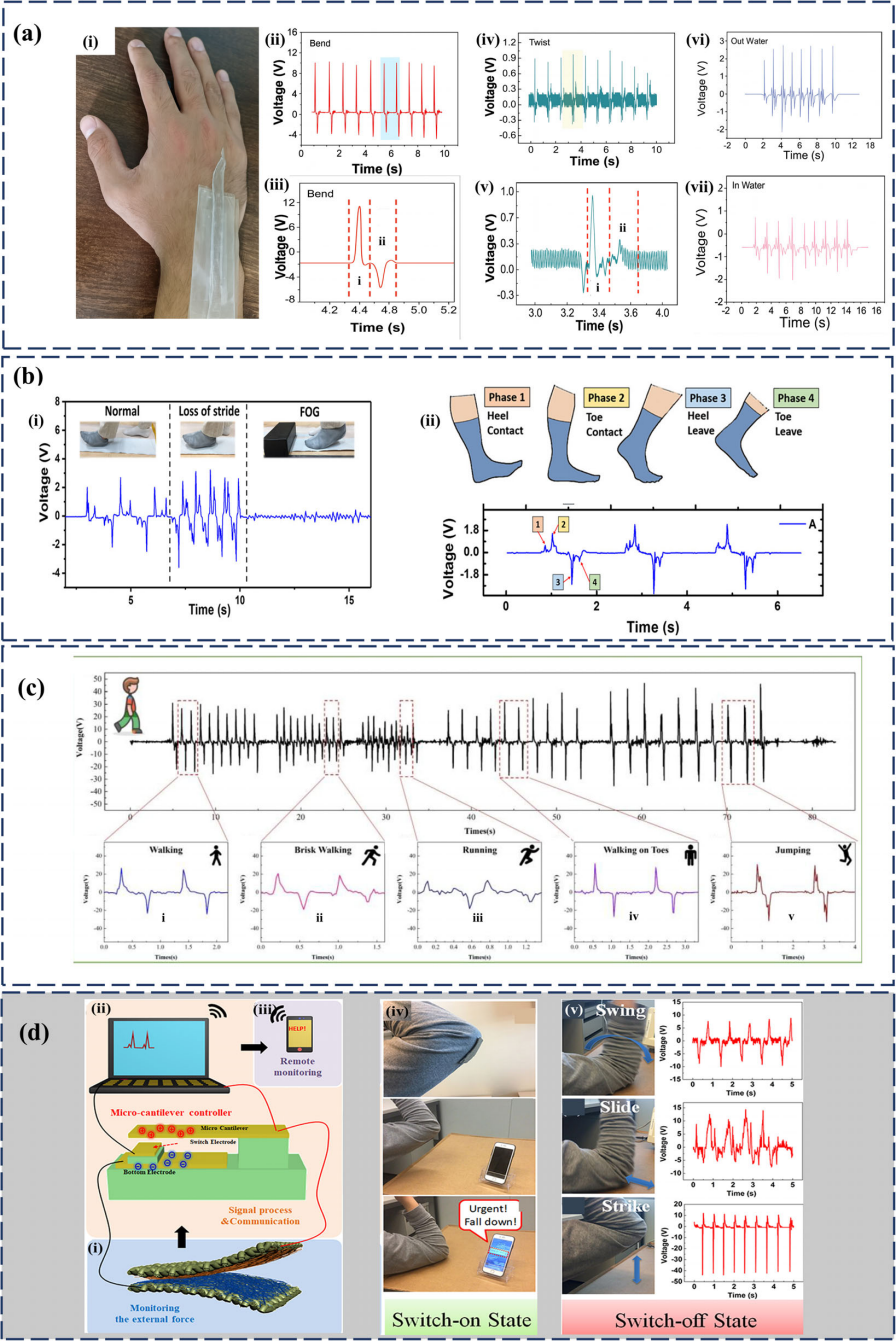
Human motion monitoring using wearable hybrid sensors. a) (i) PTSS attached to the skin without any skin irritation. (ii‐v) Wrist bending and twisting motion and their corresponding voltage response with a zoomed view of each cycle. (vi, vii). PTSS signals are out of the water and inside the water. Reprinted (Adapted) with permission.^[^ ^167^ ^]^ Copyright 2022, MDPI. b) Smart hybrid sock for detecting disability in regular walking. (i) Comparative gait analysis for regular walking and person with walking disabilities‐ loss of stride and freezing of gait (FOG). (ii) Typical walking cycle consisting of^[^ ^1^ ^]^ heel contact,^[^ ^2^ ^]^ forefoot/toe contact,^[^ ^3^ ^]^ heel leave, and^[^ ^4^ ^]^ forefoot/toe leave and its corresponding voltage output signal. Reprinted (Adapted) with permission.^[^ ^171^ ^]^ Copyright 2019, American Chemical Society. c) (i) Human posture monitoring using PT‐HNG. (ii‐vi) The output dependence on the range of physical movements. Reprinted (Adapted) with permission.^[^ ^168^ ^]^ Copyright 2019, Elsevier. d) Remote emergency fall alert microsystem. (i) Schematic of the sensing setup with PT‐HNG and micro‐cantilever. PT‐HNG served as a force detector in case of a sudden fall. (ii) PT‐HNG is connected to a micro‐cantilever, which acts as a switch for sending an emergency signal in case of a sudden fall. (iii) The emergency message was sent to a designated remote terminal. (iv) The force of the impact of a sudden fall generates an electrical pulse through the PT‐HNG. If this force generates an electrical output beyond a preset voltage value, a signal will be triggered to pull the micro‐cantilever downward the bottom electrode to activatethe computer program to send an emergency message. (v) The sensor can detect various human motions at the switch‐off stage. Reprinted (Adapted) with permission.^[^ ^172^ ^]^ Copyright 2018, Elsevier.

A PT‐HNG sport sensor was designed with PDVF as a piezoelectric layer and PTFE as the triboelectric layer, with stretchable hydrogel used as an electrode.^[^ ^167^ ^]^ The device was attachable to the human wrist (**Figure** **13**a‐i). Figure 13a‐ii presents the voltage output during the bending motion of the wrist. The wrist bending produced a sharp positive upper peak (Figure 13a‐iii‐1) and a lower opposite peak (Figure 13a‐iii‐2) generated with the straightening motion of the wrist. Figure 13a‐iv demonstrates the twisting motion of the wrist, where the twist in the internal direction is presented by Figure 13a‐iv‐1, and the twist in the external direction is denoted by Figure 13a‐iv‐1. Because of the hybrid nature of the sensing device, it could detect human motion even in underwater conditions. It was reported to produce 2.45 V output for wrist bending motion (Figure 13a‐v), which was only 1.25 V in underwater conditions due to the absence of TENG output when in contact with water (Figure 13a‐vi). Thus, the devised hybrid sensor could effectively monitor swimming and underwater diving motion. Moreover, the hydrogel electrode used with PT‐HNG had self‐healing properties, enabling it to withstand high‐strength impact. However, this stretchability will not meet highly repetitive stretchable applications as hydrogel can only be stretched to 70.11% after healing, representing degraded mechanical properties.

For monitoring walking, a PT‐HNG integrated smart sock was designed with poly(3,4‐ethylenedioxythiophene) polystyrene sulfonate (PEDOT: PSS)‐coated fabric TENG and PZT piezoelectric chips.^[^ ^171^ ^]^ The designed sock was fully wearable and demonstrated its ability for gait analysis during human walking movement when the sensor was attached to the sock's heel and toe region. Gait analysis involves a thorough assessment of individual‐specific movement patterns, which is crucial for detecting issues related to muscles, nerves, or skeletal structures, identifying the origins of pain experienced during human movement, diagnosing bone deformation or misalignments, and uncovering muscle or nerve dysfunction.^[^ ^173^, ^174^, ^175^, ^176^, ^177^ ^]^ These anomalies can cause a deviation from regular walking or running patterns. However, it is difficult to evaluate these conditions in routine clinical checkups as symptoms are irregular and primarily triggered in uncertain scenarios. Thus, the designed hybrid sock can be crucial for real‐time monitoring of potential patients. Figure 13b‐i presents a loss of strides stage with short interval cycles before the freezing of gate (FOG) stage, signified by irregular and much lower voltage output. A complete waking cycle consists of four distinguishable segments‐ heel touch, toe touch, heel leave, and toe leave.^[^ ^27^, ^28^ ^]^ Each segment produced a clear, distinguishable voltage output (Figure 13b‐ii). Even though the sensor output can vary depending on the user's weight, foot size, and walking style, the minor variation in the output did not hamper the sensor's effectiveness as the process solely relies upon waveform generation and its analysis. However, similar to the previously reported wearable sensors,^[^ ^178^, ^179^, ^180^ ^]^ the gait data produced by the hybrid sock required data analysis incorporating a machine learning algorithm due to the highly inconsistent nature of human movement.

Another PT‐HNG consisting of PZT‐embedded PDMS was utilized to capture a sequence of activities such as walking, brisk walking, running, walking on toes, and jumping, demonstrating the PT‐HNG's advanced capability and improved consistency in monitoring human posture.^[^ ^168^ ^]^ The sensor produced linear output (R^2^  =  0.99392) with excellent sensitivity (S  =  18.96 V kPa^−1^) within the 100–800 kPa pressure region. As this pressure is within the range of the pressure created due to human movement (100 kPa and 370 kPa), the sensor was an excellent candidate for Human motion monitoring. Multiple signals are recorded during the moving stage, providing detailed information about human motion based on subsequent peaks and cycle's shapes, amplitudes, and time intervals (Figure 13c‐i). Figure 13c(ii‐vi) provides enlarged views of the signals shown in Figure 13c‐i, highlighting the device's effectiveness in monitoring human motions and also, analyzing the gait, which offered detailed information about the stride frequency of different people, which can be utilized to distinguish individuals. However, a significant drawback remained as sensitivity decreased at the low‐pressure level when the input pressure was less than 100 kPa.

#### 6.2.2 Fall Alert System

Falling off while walking at home or outside is one of the major causes of injuries for older people.^[^ ^181^ ^]^ Failure to notice the incident on time may delay required medical intervention, which can result in fatal complications and possibly death for the injured person. A fast‐responding microsystem was developed by preparing a PT‐HNG with electrospun silk fibroin and PVDF for sudden fall detection without external intervention.^[^ ^172^ ^]^ A microcantilever and bottom electrode were attached to the two electrodes of PT‐HNG to harness the large electrostatic field generated from the device in case of a sudden fall. In the case of the generation of an appropriate electrostatic field, the microcantilever was pulled down to the bottom electrode, which functioned as a switch to send a warning message to the designated responsible personnel (Figure 13d(i‐iii)). The applicability of the device was demonstrated by attaching it to the wearer's elbow, and an SOS message was received immediately after knocking the elbow on a table with a large force (Figure 13d‐iv). A threshold voltage was set, corresponding to a particular force on the device, by considering Young's modulus of the micro‐cantilever and the initial gap between the relative area and dimensions of the bottom electrode and the micro‐cantilever. Below this threshold voltage, no emergency message was sent, but the sensor can still operate the motion monitoring device in the switch‐off stage (Figure 13d‐v). However, depending on the tripping position and types, sudden pressure can occur not only in the elbow position but also in various body parts.^[^ ^182^, ^183^ ^]^ Thus, the designed system needs to be attached to multiple body positions. Based on the output generated, a predictive model needs to be implemented to realize the fall alert system in smart clothing successfully.

#### 6.2.3 Self‐Powered Weather Monitoring

**Figure 14:**
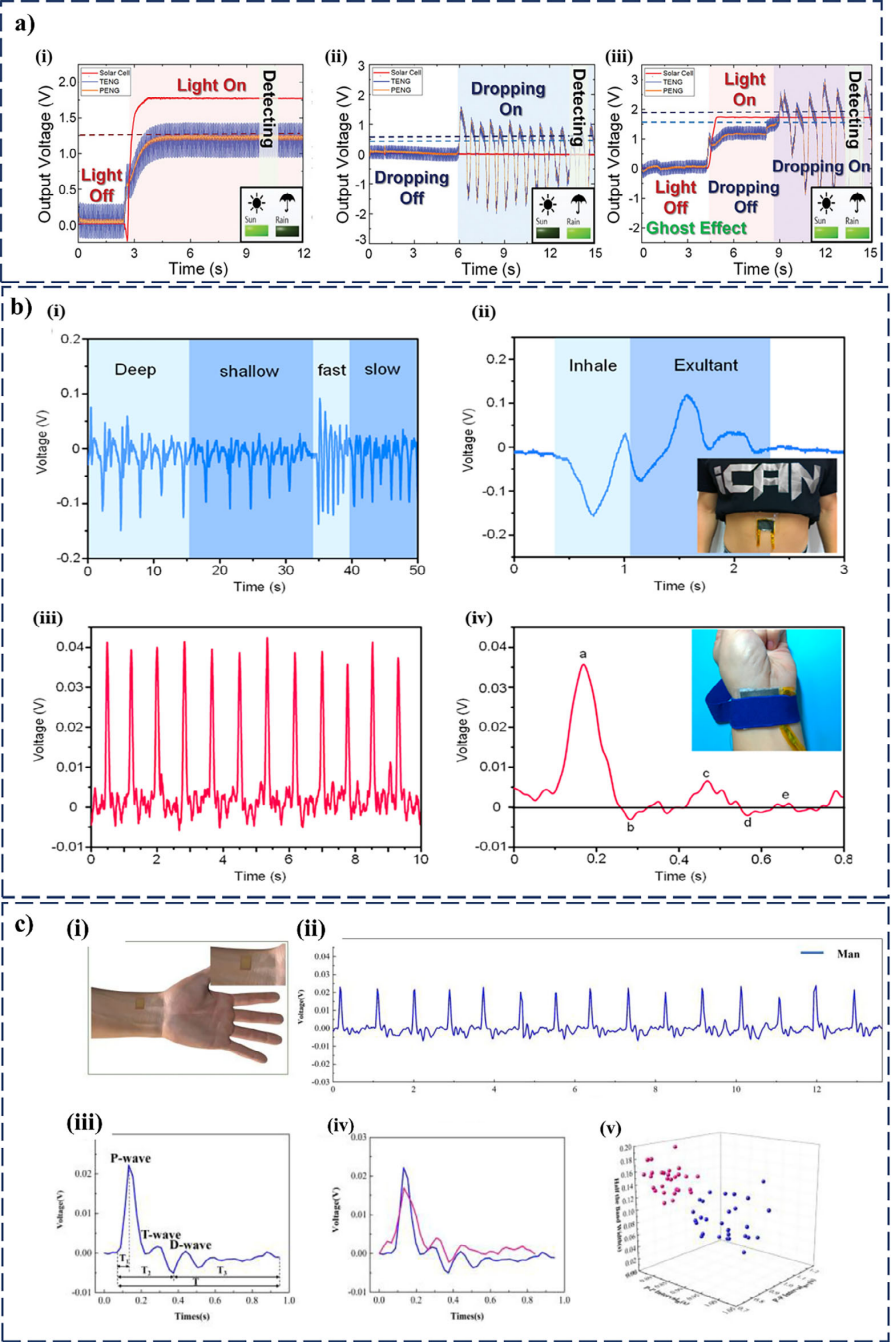
a) FPT‐HNG for self‐powered weather monitoring i) sunny day, ii) rainy day, and iii) both sunny and rainy day. Reprinted (adapted) with permission.^[^ ^186^ ^]^ Copyright 2021, John Wiley and Sons. b) PT‐HNG for respiration and artery pulse monitoring. (i) Respiration signal for different states of breathing. (ii) Zoomed‐in view of a breathing cycle. (iii) Artery pulse signal (iv) Zoomed‐in view of an artery pulse cycle. Reprinted (Adapted) with permission.^[^ ^169^ ^]^ Copyright 2017, Elsevier. c) Continuous blood pulse wave sensing with PT‐HNG. (i) Schematic diagram of HPTS attached to the wrist. The HPTS was positioned over skin and muscle but was sensitive enough to detect the pulse motion, harnessing the slight movement of the radial artery. (ii) Continuous pulse wave generation for a healthy man. (iii) Zoomed‐in view of each pulse cycle. (iv,v) Comparative analysis of P wave, conducted between a healthy man and a woman. Reprinted (Adapted) with permission.^[^ ^168^ ^]^ Copyright 2019, Elsevier.

A significant portion of self‐powered weather monitoring systems research primarily relies on EMG and TENG. These technologies are employed to harness ambient mechanical energy from the environment, such as wind or vibrations, and convert it into electrical power.^[^ ^184^, ^185^ ^]^ However, flexible PT‐HNG has been reported to be employed as a weather monitoring sensor, combining a transparent TENG and a flexible PENG with a solar cell.^[^ ^186^ ^]^ The three‐layer structure consisted of FEP film (triboelectric part), middle silicon layer (solar cell), bottom BaTiO_3_ nanoparticles, and MWCNT (piezoelectric part). The sensor could detect the status of sun and rain in an open environment, with an HNG acting as a high‐performance rain sensor while the solar cell was implemented as a light sensor. To ensure reliable operation in high‐humidity or rainy environments, the device was encapsulated with PDMS, providing excellent waterproofing and environmental stability. A signal system was developed to indicate weather status when output exceeds certain thresholds autonomously. The threshold voltage for the sunlight activation condition was set at 1.25 V (**Figure** **14**a‐i). Without sunlight activation, the threshold voltages of 0.6 V for TENG and 0.4 V for PENG were established (Figure 14a‐ii). However, a ghost effect occurred due to the solar cell, creating a large DC output when light and rain were available simultaneously in the sensing environment (Figure 14a‐iii). Thus, under both sunlight and rain activation, the threshold voltages for TENG and PENG were adjusted to 1.8 V and 1.6 V, respectively, for successful monitoring of the weather conditions. Even though the sensor demonstrated great promise in autonomous weather monitoring, its application can be further broadened to monitor air quality in rainy conditions by measuring the acidity of rainwater through the triboelectric effect, which will provide a holistic understanding of the weather in a particular place.

#### 6.2.4 Bioelectronics

Bioelectric wearable sensors are essential for continuously monitoring various physiological parameters, providing valuable insights into an individual's health and well‐being.^[^ ^187^, ^188^, ^189^ ^]^ These sensors offer the flexibility of long‐term monitoring outside clinical facilities, enabling individuals to take charge of their health to detect anomalies and potential health issues and make timely decisions based on personalized data. Thus, they are vital in proactive healthcare management and enhancing the overall quality of life. Various bioelectronics sensing devices encompassing HNGs have been reported for monitoring vital signs such as heart rate, radial artery wave, respiration, and coughing.^[^ ^168^, ^169^, ^170^, ^186^, ^190^, ^191^ ^]^

A flexible PT‐HNG has been reported to integrate PVDF‐TrFE as a piezoelectric layer and micropatterned PDMS as a triboelectric layer.^[^ ^169^ ^]^ The sensor demonstrated sensitivity of 7.84 and 1.42 V N^−1^, corresponding to TENG and PENG. The sensor was implemented as a wearable e‐skin because of its smaller and lightweight form factor, allowing discreet and unobtrusive placement on the body, enhancing user comfort and usability. The device as placed on the belly of a user to monitor human respiratory rate and depth (Figure 14b(i‐ii)). The sensor also provided information about respiration depth and cycle, which are closely related to the user's physiological state. Arterial pressure is another critical physiological parameter that provides essential patient cardiovascular health data. Remote Arterial Pressure (RAP) tracking with wearable sensors enables healthcare providers to continuously monitor a patient's blood pressure and rapidly detect any sudden changes or abnormalities crucial in managing critical cardiovascular conditions, such as hypertension or hypotension. Ten seconds of RAP was reported for a 23‐year‐old woman, which read a pulse frequency of 72 beats per minute (Figure 14b(iii‐iv)).

Another PT‐HNG was reported for RAP monitoring where microstructure was imparted on the nonpolarized PZT‐PDMS composite layer, while polarized PZT‐PDMS acted as a piezoelectric layer.^[^ ^168^ ^]^ The PT‐HNG achieves a sensitivity of 15.43 V/kPa through the triboelectric effect in the pressure range of 0–100 kPa, while sensitivity increases to 18.96 V kPa^−1^ in the case of 100–800 kPa pressure, utilizing both piezoelectric and triboelectric effects, proving the improved sensitivity due to the hybrid structure.^[^ ^168^ ^]^ For the excellent linearity, the fast response time (45 ms–85 ms), and high sensitivity, it was possible to detect and analyze slight vibration from the radial artery as a pulse wave from the wrist of a healthy man (Figure 14c(i‐ii)). The sensitivity of the sensor was large enough to capture the essential physiological data such as pulse signal‐ percussion wave (P‐wave), the tidal wave (T‐wave), and the diastolic wave (D‐wave), which are related to the heart rate, ventricular pressure, systolic blood pressure, and diastolic blood pressure (Figure 14c‐iii). The P‐wave is the peak arterial pressure, regulated by cardiac output, ventricular ejection velocity, and arterial compliance. A comparative study of the P‐wave of a healthy man and woman revealed the stronger P‐wave observed in a man due to greater ventricular ejection velocity (Figure 14c(iv,v)). The T‐wave provides data about vascular elasticity and peripheral vascular resistance. Lastly, the D‐wave, a minor wave, provides insights into cardiac function and blood flow status. Although there was a loss of sensitivity, and triboelectric became dominant at a pressure below 100 kPa, the sensor remained relevant for practical applications.

## 7 Challenges, Future Prospects of Developing PT‐HNGs, and Conclusion

Nanogenerators have garnered significant attention due to their potential in energy harvesting and self‐powered sensing applications. Their miniaturized size, lightweight nature, high sensitivity, and durability make them promising candidates for advancing research frontiers and a wide range of commercial applications. A particularly compelling strategy for enhancing the performance of these devices involves the integration of multiple energy conversion mechanisms (two or more) into a single system. Creating a synergetic effect to further boost the piezoelectric and triboelectric output can further leverage the hybridization of nanogenerators.

Despite these advantages, several challenges persist in developing and deploying PT‐HNGs. One major limitation lies in system integration, where device miniaturization, efficient signal transmission, and complex charge alignment control in hybrid architectures remain key difficulties. In addition, durability issues such as ensuring long‐term performance, encapsulation from the external environment, improved moisture and thermal regulation, and signal stability continue to hinder practical use. The heterogeneous integration of diverse material types in hybrid systems often results in mechanical mismatch, interfacial delamination, or performance degradation under repeated stress cycles, compromising device reliability.

From the enhanced output perspective, achieving high open‐circuit voltage, short‐circuit current, and power density, along with improved sensitivity, enhanced precision and broader detection range, remains a significant goal. However, optimizing output performance is often constrained by trade‐offs between material flexibility and electrical properties, particularly in wearable applications. Furthermore, electronic compatibility issues, such as the need for reliable interconnections, efficient rectification, and integration with wireless transmission for IoT, present additional design complexity. High impedance mismatch and nonlinear charge flow behavior further complicate signal conditioning and real‐time processing. Looking ahead, the continued evolution of hybrid nanogenerator technology hinges on overcoming these integration and miniaturization challenges. Future research is expected to focus on advanced micro/nanofabrication techniques, which could facilitate the development of more compact, integrated structures without sacrificing performance. At the same time, enhancing long‐term stability through encapsulation strategies using stretchable, self‐healing, or environment‐resistant polymers will be critical. Machine learning and artificial intelligence (AI) assisted modeling may also offer new pathways to optimize device architectures, predict long‐term reliability, and enable adaptive signal processing in multifunctional sensing platforms.

**Figure 15:**
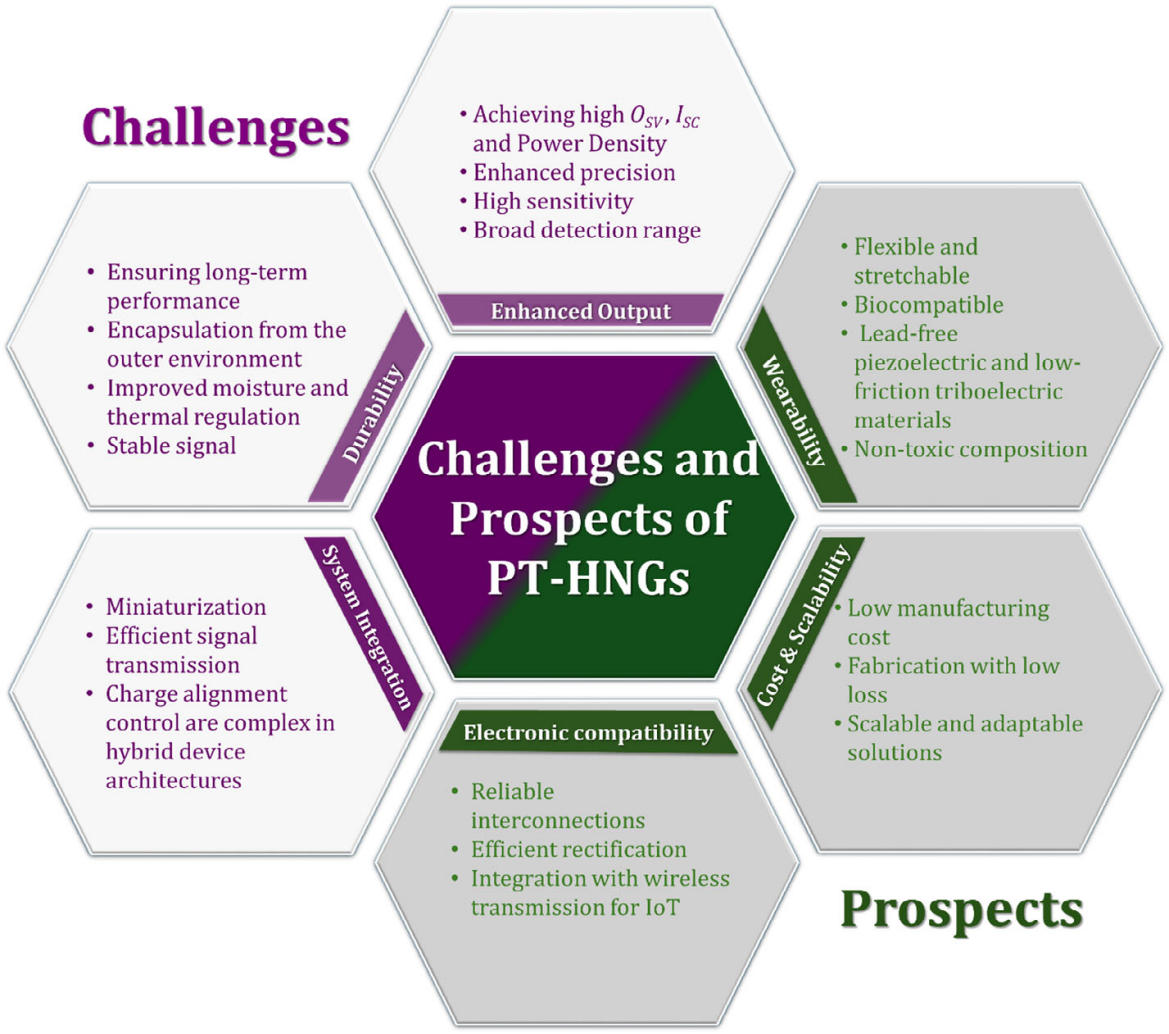
Illustration of challenges and prospects for PT‐HNGs development.

In terms of future prospects, materials offering wearability, including flexibility and stretchability, biocompatibility, non‐toxicity, and the use of lead‐free and low‐friction triboelectric layers, will be pivotal in wearable applications. Additionally, the cost and scalability advantages, such as low manufacturing cost, low‐loss fabrication, and scalable and adaptable solutions, further strengthen the commercial viability of PT‐HNGs. Developing hybrid materials, such as PVDF blended with graphene, MXenes, or other functional nanofillers, offers new opportunities for tuning dielectric, mechanical, and surface properties for improved energy harvesting and sensing. Moreover, integrating PT‐HNGs with emerging technologies such as soft robotics, smart textiles, or implantable biosensors opens new frontiers in real‐time physiological monitoring, healthcare diagnostics, and human‐machine interfaces. Furthermore, research is shifting toward all‐in‐one multifunctional devices that not only harvest energy but also monitor multiple stimuli (e.g., pressure, strain, temperature, biochemical signals), thereby reducing the need for external power sources and additional sensors. These multifunctional PT‐HNGs will be crucial for realizing truly autonomous and self‐sustaining wearable systems. As these challenges are progressively addressed, hybrid systems are expected to play a pivotal role in the next generation of energy harvesting and self‐powered sensing technologies. **Figure** **15** summarizes the major challenges and prospects for PT‐HNGs development.

In conclusion, while HNGs present a promising pathway for maximizing energy conversion efficiency, their widespread adoption is contingent upon addressing the challenges associated with device complexity and size. The future development of these systems will require a multidisciplinary approach, combining advances in materials engineering, device fabrication, and circuit integration. With sustained research efforts and strategic innovations, hybrid nanogenerators are well‐positioned to revolutionize energy harvesting and sensing applications, paving the way for the next wave of sustainable, self‐powered technologies.

## Conflict of Interest

The authors declare no conflict of interest.
